# Material recognition from cross-category morphing

**DOI:** 10.1038/s41598-026-59920-x

**Published:** 2026-10-01

**Authors:** Chenxi Liao, Filipp Schmidt, Jacob R. Cheeseman, Masataka Sawayama, Roland W. Fleming, Bei Xiao

**Affiliations:** 1 Department of Neuroscience, American University, Washington DC, USA; 2 Department of Computer Science, American University, Washington DC, USA; 3 Justus Liebig University Giessen, Giessen, Germany; 4 Center For Mind, Brain and Behaviour (CMMB) of the Universities of Marburg, Giessen and TU Darmstadt, Marburg, Germany; 5 Graduate School of Information Science and Technology, Hokkaido University, Sapporo, Japan; 6 Prometech CG Research, Tokyo, Japan

**Keywords:** Materials science, Psychology, Psychology

## Abstract

Materials from distinct categories share common image features but also exhibit unique characteristics. How do humans recognize materials despite their enormous appearance variations? Using generative neural networks, we create both prototypical and ambiguous materials that morph between categories. To capture the richness of material representation, we characterize how people judge materials using cross-material morphs through three behavioral tasks: material categorization, material property rating, and visual similarity judgment. We find that morphing smoothly modulates perceptual scales of material appearance. Despite this smooth variation, participants can reliably identify the prototypical materials within given appearance ranges, indicating a strong association between material categories and their visual characteristics. Material properties structure the perceptual space, where the salient dimensions–particularly rigidity–strongly correlate with perceived material categories. In contrast, analyzing image embedding derived from a self-supervised deep learning model reveals that learning visual similarities alone is insufficient to reproduce human perceptual space of materials. Together, our results suggest that material representation is more than visual similarities and may require learning material properties that structure relationships among materials within and across categories. Visual features might be used flexibly and task-dependently to support multiple levels of material perception, ranging from discrimination to material property inference to categorization.

## Introduction

Crystals and candies may look alike in many ways, but recognizing that they are made of different physical materials would have significant consequences for decision-making, such as whether to consume them. Categorization refers to “the process of mapping continuous sensory inputs onto discrete, behaviorally relevant concepts”^1^, allowing us to store, structure, and communicate about our environment^2–4^. While extensive work has examined how we categorize faces, objects, and scenes^5–7^, far less is known about how we categorize materials from visual appearance^8,9^. Yet material categorization is a fundamental ability that guides our interactions with the physical environment^10^.

**Fig. 1:**
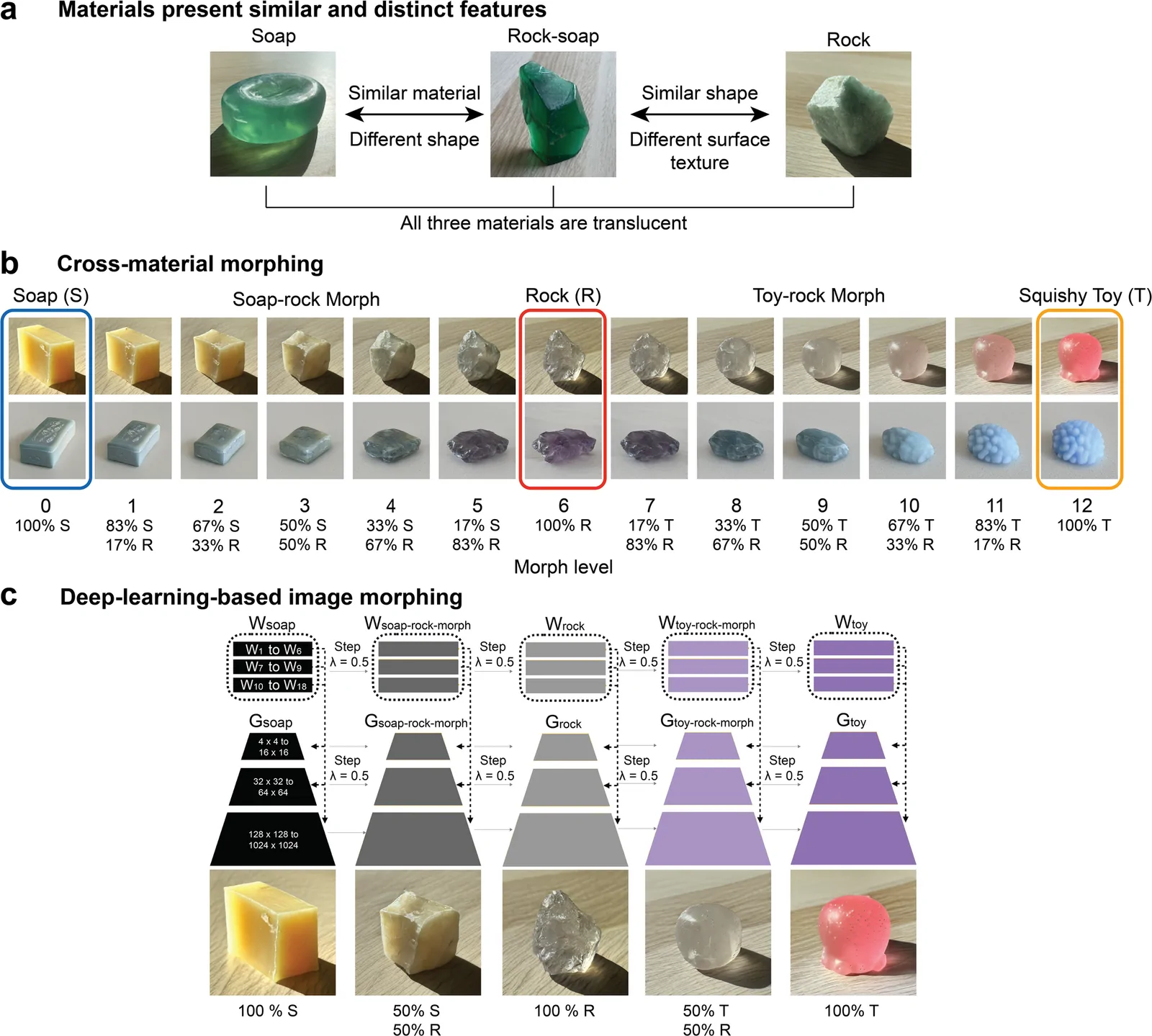
Cross-material morphing via deep generative models. (**a**) Materials present both similar and distinct features. The examples show photographs of a soap (Leftmost), a rock (Rightmost), and a rock-soap (Middle)—a soap made to mimic the appearance of a rock. (**b**) Image morphing allowed the transition of visual appearance across prototypical materials. Intermediate images (coded from 0 to 12) were a mix of a pair of prototypes: soap (100%S), rock (100%R), and squishy toy (100%T). (**c**) Cross-material image morphing system. All images were generated from the deep generative model StyleGAN2-ADA. With the corresponding latent code and generator, the pre-trained StyleGANs generate the images of prototypical materials: soaps ($w_{soap}$ and $G_{soap}$), rocks ($w_{rock}$ and $G_{rock}$), and squishy toys ($w_{toy}$ and $G_{toy}$). Linear interpolation between these models produces the morph material images. The step size, *λ*, indicates the strength of morphing with the target material. For example, at *λ = 0.5*, a morphed image is produced from the interpolated latent code (e.g., $w_{soap-rock-morph}$) and generator (e.g., $G_{soap-rock-morph}$), indicating a combination of 50% soap and 50% rock.

In everyday life, we encounter a vast array of materials with highly diverse visual appearances. Categorizing materials from appearance is challenging because material appearance is inherently multidimensional. Materials are jointly defined by various visual features, and the mapping between category and visual features is complex: Physically different materials can exhibit similar visual features, such as color, glossiness, or translucency, while physically similar materials can appear visually distinct (Fig. 1a). Despite this complexity, humans can rapidly recognize materials and categorize them into familiar categories^11^. Beyond categorization, we also extract semantic properties of materials from appearance^12–18^, and previous work has shown that perceived properties and perceived category are closely linked^19–22^. Characterizing material categorization and property inference, and how each relates to visual appearance, therefore offers insight into how the brain represents complex, multidimensional stimuli to interpret the world around us.

Previous studies of material categorization mostly focused on highly controlled stimuli that are typically varied along a single dimension, such as specific physics-based model parameters or certain image features related to physical properties (e.g., specular highlight structure). In these studies, factors such as shape, optical and mechanical properties are typically manipulated independently ^13,21,23–26^. This controlled approach helps discover the relationship among image features, physical parameters, and material categories. However, such an approach artificially removes the natural co-variation among the factors, thereby forcing a direct mapping of certain visual features and perceived categories. Furthermore, it typically relies on strong assumptions about which factors to manipulate, thereby further biasing the inferred mappings. In real-world settings, by contrast, material appearance arises from the joint interaction of many co-varying visual features, and this natural combination of features is likely to shape both material categorization and estimates of material properties.

Here, we investigated material perception under conditions in which visual appearance and material properties co-vary. More specifically, we ask: How do humans categorize and represent materials from the continuous multidimensional variation in visual appearance? To address this question, we trained generative neural network models on photos of different materials, and used them to create morph continua of visual appearance that seamlessly transition between prototypical categories: soaps, rocks, and squishy toys ^22,27^. The resulting morph continua reflect the statistical structure of these real-life materials, with multiple material features varying simultaneously, including shape, color, texture, and other material properties (see Fig. 1b). Cross-material morphing elicits vivid impressions of object-shaped materials and enables the creation of ambiguous materials that blend features from different material categories, making them particularly valuable for probing the nuanced relationship between visual appearance, perceived category, and material properties.

Using these cross-material morphs, we examined how humans judge materials from the continuous variation of appearance, seeking to characterize how material categorization relates to material property ratings and visual similarity. To foreshadow the results, we found that participants reliably identified the prototypical materials from particular appearance ranges in the morph continua. A self-supervised deep learning model further implies that learning visual similarity alone is insufficient to reproduce the human perceptual embedding of materials. Instead, human material representation may rely on understanding meaningful material properties that structure the relationships among materials.

**Fig. 2:**
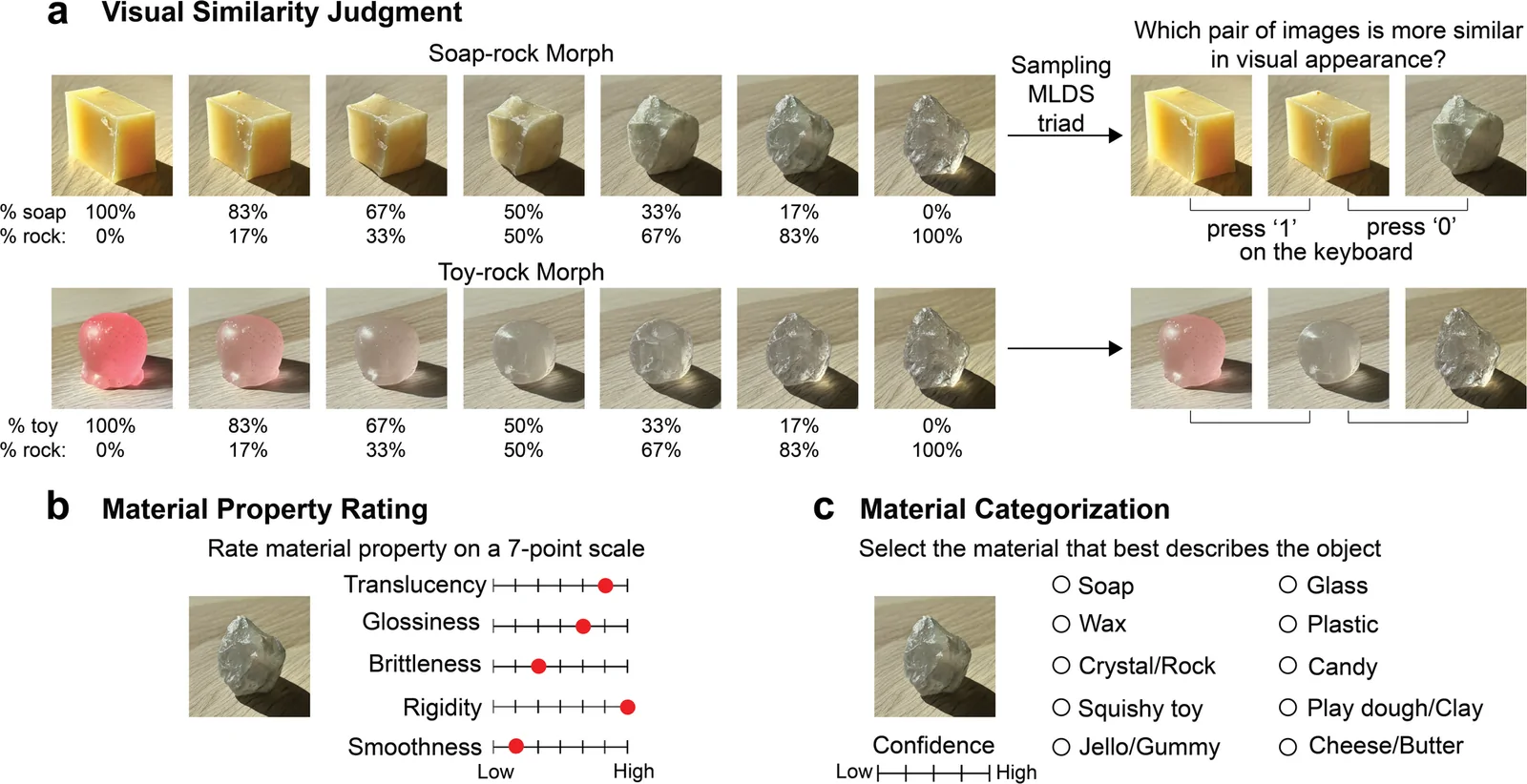
Psychophysical experiments (**a**) Visual Similarity Judgment. The MLDS triads were independently sampled from seven-level soap-rock or toy-rock morph continuum. With each triad, participants selected the pair of images that is more similar in their visual appearance. (**b**) Material Property Rating. Participants rated five material properties: translucency, glossiness, brittleness, rigidity, and smoothness on a seven-point scale. (**c**) Material Categorization. Participants chose the material category that best describes the image and then rated the confidence level of their judgment on a five-point scale.

## Morphing smoothly modulates perceptual scales of material appearances

Morphing and feature mixing between prototypical samples have long been effective approaches for generating stimuli that probe how humans represent continuous variation in visual inputs^28–30^. We developed a stimulus creation method that uses style-based deep generative models to construct cross-material morph continua between prototypical materials, soaps, rocks, and squishy toys (Fig. 1c)^22^. These materials are commonly encountered in everyday experience and span distinct optical (e.g., glossiness, translucency) and mechanical properties (e.g., rigidity). At the same time, they share features such as color and shape. This combination allows us to capture variation across both unique and shared material characteristics. By interpolating between trained models, we created 13-step material appearance morphing (level 0 to 12), progressing from a prototypical soap (level 0) to a rock (level 6), and then to a squishy toy (level 12) (see Fig. S1 for all images). The images near the morph midpoints (e.g., 50% Soap-50% Rock) possess visual features from both endpoint materials, eliciting perceptual ambiguity in material categories. We excluded the morphs between soaps and squishy toys because their intermediate morph images exhibited a limited range of variation in appearance due to the similarity of the two endpoint materials.

**Fig. 3:**
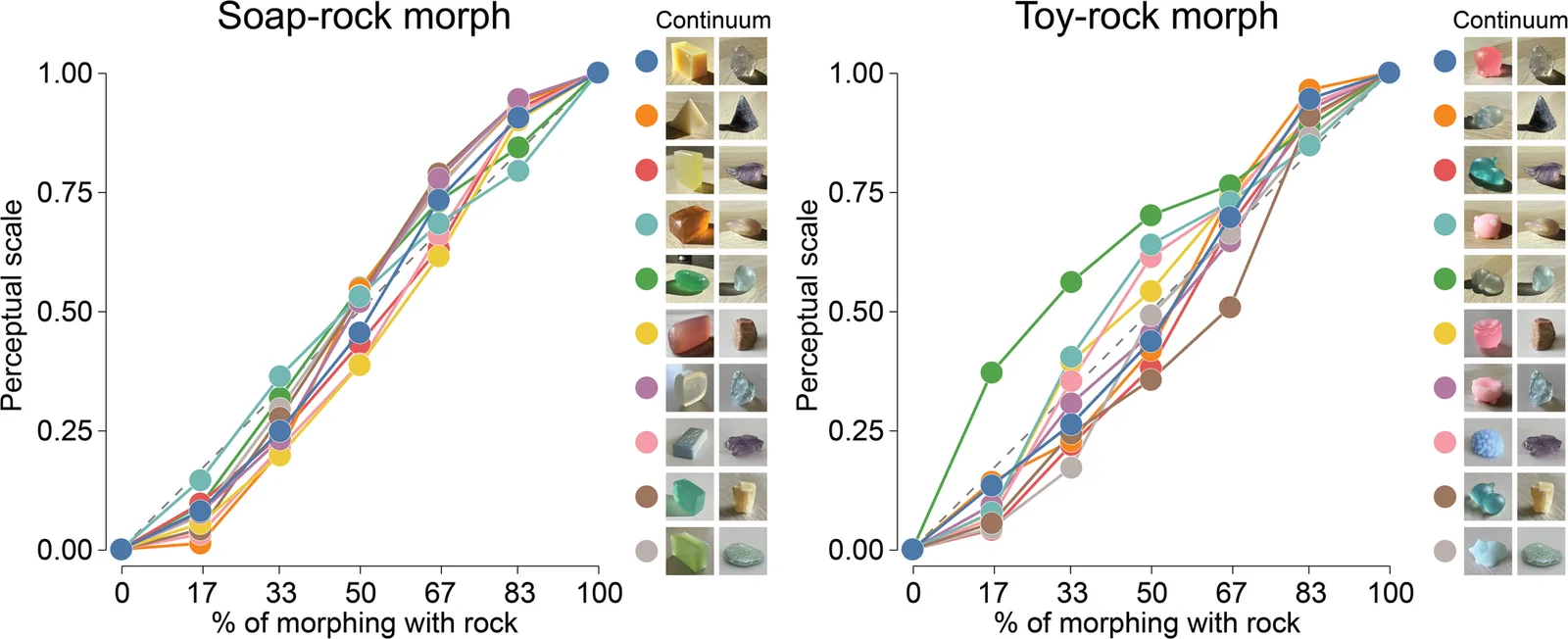
Perceptual similarity varies smoothly along morph continuum. In each panel, the solid line shows the estimated perceptual scale averaged across all participants for a seven-level morph continuum. The x-axis represents the morph level, with higher values corresponding to a greater proportion of the target rock image in the morph (see example in Fig. 2A). The gray dotted diagonal line represents the linear variation of the perceptual scale. Left: Soap-rock morph continua (morphing from soap to rock); Right: Toy-rock morph continua (morphing from squishy toy to rock). Image icons mark the endpoint images for each continuum.

We first examined the extent to which humans discriminate material appearances along the morphing continuum. To test this, in Experiment 1, we measured participants’ sensitivity to visual appearance differences along each single seven-level morph continuum (either soap-rock morph or toy-rock morph), using a triad variant of maximum-likelihood difference scaling (MLDS)^31^. In a triad, participants (N=20) were presented with three images and indicated whether the pair on the left or right appeared more similar (Fig. 2a). MLDS estimates how the perceived visual appearance changes as a function of morph level. Across all continua, the estimated perceptual scale increased monotonically and approximately linearly with morph level (linear fit $R^2$, mean = 0.972, s.d. = 0.015) (Fig. 3, see Fig. S6 for individual MLDS curves). This shows that participants could readily discriminate among variations in visual appearance and that morphing nearly uniformly modulates perceptual scales of material appearance. These near-uniform perceptual changes confirm that our morphing method continuously and predictably modulates the visual similarity of material appearance along each continuum.

## Perceptual material categories emerge from morph continua

Given the smooth variation of visual appearances, we now characterize material representation by measuring how humans categorize materials and estimate their properties. With ten diverse soap-rock-toy continua, a new group of participants (N=27) performed two tasks: Material Property Rating (Experiment 2) and Material Categorization (Experiment 3) (see Fig. 2b and 2c for experiment interface). In the Material Categorization task, participants classified each image into one of ten possible material categories and rated their confidence. Importantly, participants were not informed of the material categories used to generate the stimuli. This allowed us to test whether novel images — combining features from the three prototype categories — would be perceived as belonging to the prototypical categories or as entirely different materials. In the Material Property Rating task, participants assessed: two mechanical properties (rigidity, brittleness), two optical properties (translucency, glossiness), and a visual-tactile property (smoothness), which have been found useful in characterizing the mental representation of materials^19,22,32^. Comparing categorization judgments with material property estimations allowed us to test whether the same image would be interpreted as having different physical characteristics if it was also seen as belonging to a different category.

**Fig. 4:**
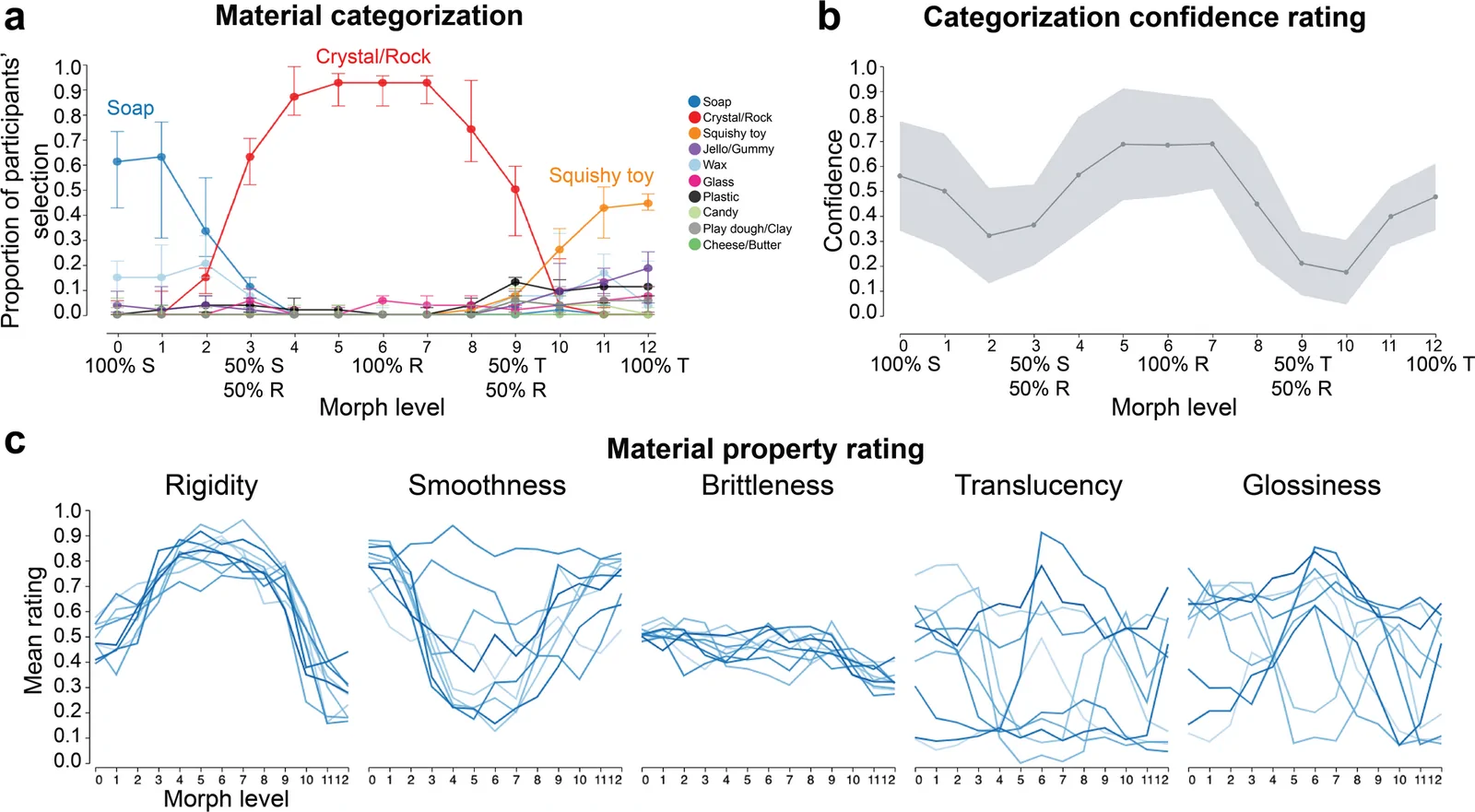
Material categorization over morph continua of prototypical and ambiguous materials. (**a**) Material categorization judgments along the morph levels. Dots represent the median proportion of participants selecting a given material at each morph level, with the error bar indicating the interquartile range (between the first and third quartile) across the 10 images at a morph level. (**b**) Modulation of confidence rating. Dots represent the median of the averaged self-reported confidence rating (normalized to 0 to 1 range) at each morph level, with the shaded region showing the standard deviation across morph continua. (**c**) Modulation of material property rating. In each panel, each individual line represents the mean property rating (normalized to 0 to 1 range) of images from a unique soap-rock-toy morph continuum.

Participants identified prototypical material categories from the cross-category morphs. Fig. 4a plots the proportion of participants selecting a specific category across morph levels. Each dot represents the median proportion of participants selecting a specific material at a given morph level, across the 10 images from all morph continua. Participants recognized images of prototypical materials (100% Soap, 100% Rock, and 100% Squishy toy) as soaps, rocks, and squishy toys, with higher agreement for crystals/rocks (median = 92.6%) compared to soaps (median = 61.1%) and squishy toys (median = 44.4%). Plateaus in the category tuning curves show that adjacent images near the morph endpoints were also recognized as prototypical materials (e.g., rock). Images of prototypical materials were sometimes misperceived as other materials. For example, prototypical soaps were perceived as wax 17% of the time, prototypical rocks were perceived as glass 5% of the time, and prototypical squishy toys were perceived as jello/gummy 16% of the time (see Fig. S3 for the distribution of selected categories). These results demonstrate that participants group a range of stimuli into the same material category along morph continua despite their differences in appearance, indicating clear associations between specific appearances and familiar material categories.

Image morphing systematically modulated perceived material categories and confidence. As shown in Fig. 4a, category judgments shifted gradually along the morph continua, with abrupt transitions near the midpoint (e.g., a sharp increase in “Crystal/Rock” judgment from 67% Soap-33% Rock (morph level 2) to 50% Soap-50% Rock (morph level 3), a sharp drop from 50% Squishy toy-50% Rock (morph level 9) to 67% Squishy toy-33% Rock (morph level 10). Notably, participants often perceived the intermediate morph images as materials different from either prototype, especially morphs between rock and squishy toy. For example, at 67% Squishy toy-33% Rock, participants categorized the stimuli as wax, play dough, jello/gummy, and plastic. Meanwhile, participants’ confidence ratings were significantly lower at morph midpoints (50% Soap-50% Rock and 50% Squishy toy-50% Rock) than at prototypical endpoints (all *p < 0.001* with Kruskal–Wallis test) (Fig. 4b).

## Space of material properties clusters materials into perceptual categories

Cross-material morphing modulates the perceived material properties in distinct ways. Fig. 4c shows the mean material property ratings for the tested morph continua. For each property, we fitted a linear mixed model to characterize how morph level modulated perceived property, with morph continuum and participants as random effects. Rigidity and smoothness showed the clearest and most consistent linear modulation along the morph levels. Perceived rigidity increased (monotonically) from soap to rock (*β = 0.358, SE = 0.052, t = 6.859, p < 0.001*) and decreased from rock to squishy toy (*β = -0.578, SE = 0.048, t = -12.142, p < 0.001*), closely tracking the material categorization pattern. Smoothness followed the opposite trajectory, decreasing from soap to rock (*β = -0.403, SE = 0.088, t = -4.591, p < 0.001*) and increasing from rock to squishy toy (*β = 0.318, SE = 0.086, t = 3.687, p < 0.01*). These patterns indicate that rock-like materials were consistently perceived as harder and rougher than the other categories. In contrast, optical properties varied less systematically along the continua. Glossiness showed no linear trend from soap to rock (*β = 0.075, SE = 0.116, t = 0.643, p = 0.535*) but decreased near-monotonically from rock to squishy toy (*β = -0.254, SE = 0.110, t = -2.306, p < 0.05*). Translucency showed no consistent linear modulation and varied depending on the particular images (soap-to-rock: *β = -0.101, SE = 0.113, t = -0.891, p = 0.396*; rock-to-toy: *β = -0.065, SE = 0.110, t = -0.593, p = 0.567*). Lastly, brittleness showed little overall variation, which remained stable from soap to rock (*β = -0.050, SE = 0.056, t = -0.895, p = 0.378*) and decreased only marginally from rock to squishy toy (*β = -0.135, SE = 0.072, t = -1.885, p = 0.070*), likely reflecting limited variation in this property across our stimulus set.

To quantify the relationship between perceptual categories and properties, we constructed image-by-image Representational Dissimilarity Matrices (RDMs) and conducted representational similarity analysis (see supplementary Fig. S9). The material categorization RDM most strongly correlated with the rigidity RDM (mean *r = 0.672*, 95% CI [0.663, 0.682] in bootstrapping, *p < 0.001*). In contrast, the material categorization RDM showed overall low-to-moderate correlation with smoothness (mean *r = 0.273*, 95% CI [0.249, 0.294] in bootstrapping, *p = 0.001*) and brittleness RDMs (mean *r = 0.204*, 95% CI [0.185, 0.221] in bootstrapping, *p = 0.001*), and only minimal correlations with translucency (mean *r = 0.057*, 95% CI [0.038, 0.078] in bootstrapping, *p = 0.009*) and glossiness RDMs (mean *r = 0.107*, 95% CI [0.086, 0.126] in bootstrapping, *p = 0.001*). Together, these findings indicate that morphing tuned perceived mechanical properties—especially rigidity—in ways that closely aligned with material categorization, while optical properties varied more inconsistently across categories.

**Fig. 5:**
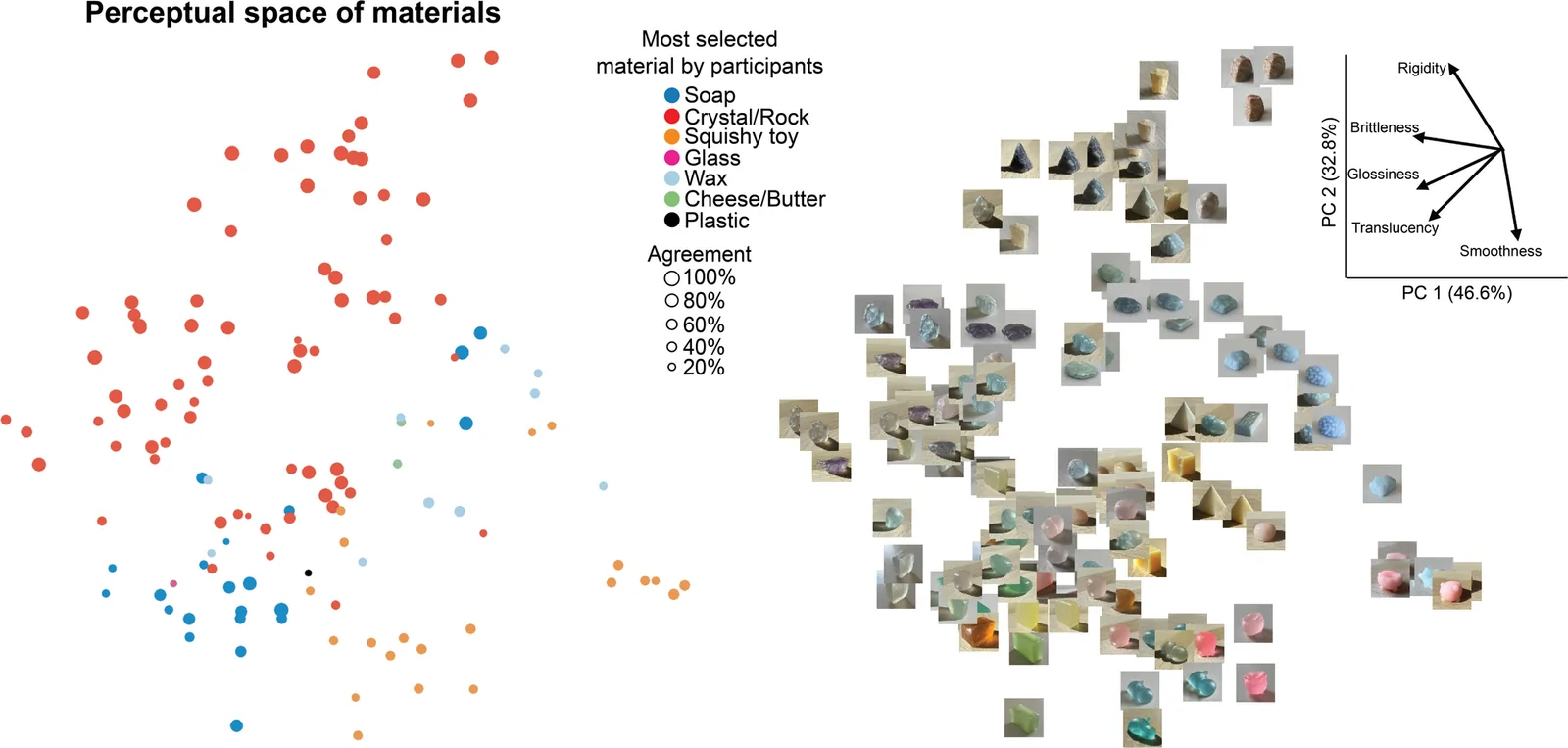
Perceptual space of materials. PCA of 130 images used in the psychophysical experiments based on the mean material property ratings. Left: The dot color represents the most frequently chosen material category for an image, while the dot size indicates the level of agreement among participants for choosing such a category. Right: The same PCA, annotated by corresponding images. The top right corner shows loadings for five material properties.

We further depict the perceptual space of materials by applying Principal Component Analysis (PCA) to the material property ratings. Fig. 5 shows the distribution of 130 images in the perceptual space defined by the first two principal components (PCs) derived from the five mean property ratings. We then annotated the PCA with the material categorization results. The color of the dots represents the most frequently selected material category for each image, while the dot size indicates the level of agreement among participants in selecting that category. In the PCA, rigidity and surface smoothness emerged as the primary properties separating hard and soft materials, while these two properties were strongly anticorrelated (Kendall rank correlation *r = -0.47, p<0.001*). Images categorized as “Crystal/Rock” (red dots) were well separated from “Soap” and “Squishy toy” (blue and orange dots). Glossiness and translucency were almost orthogonal to rigidity (Kendall rank correlation *r = 0.19, p<0.001* between glossiness and rigidity; *r = -0.02, p=0.696* between translucency and rigidity). The translucent stimuli, which include both rocks and soaps, were clustered in the lower left side of the space, while the opaque stimuli, which include both rocks and squishy toys, were clustered on the right side. Moreover, some images perceived as belonging to different categories overlapped in the PCA space (middle region on PCA), indicating that certain materials can share similar properties despite being categorized differently. In sum, these results suggest that material properties structure the perceptual space of materials: mechanical properties (e.g., rigidity) define the primary axis of categorical separation, while optical properties (e.g., glossiness, translucency) capture appearance variation shared across categories.

## Characterizing material representation through learning visual similarity

**Fig. 6:**
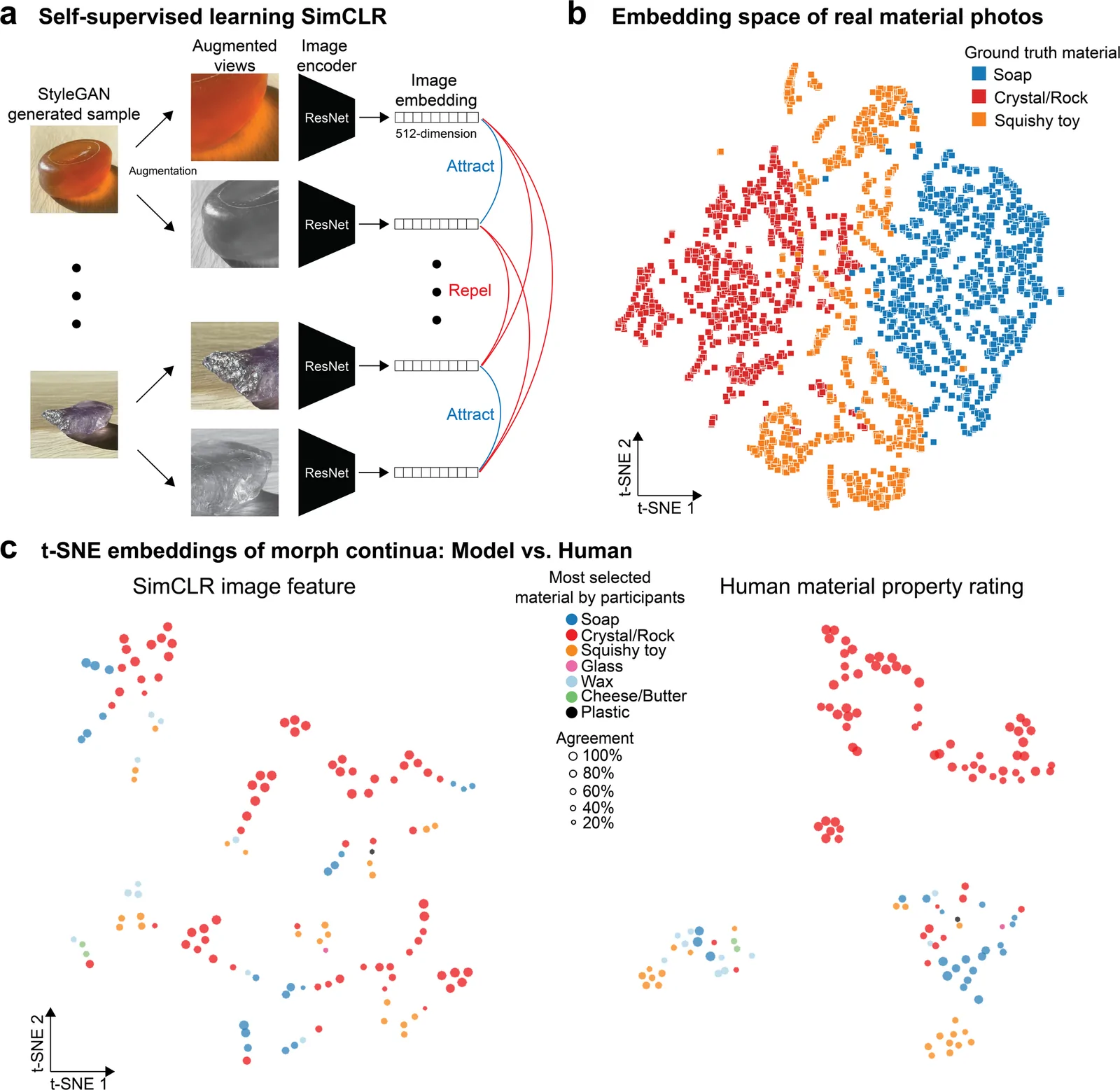
Image feature embedding of material appearances. (**a**) Self-supervised learning of material appearances with SimCLR. (**b**) Visualization of SimCLR embedding of real-world material photos with t-SNE. Three major clusters emerged: crystal/rock on the left, squishy toy in the middle, and soap on the right. (**c**) t-SNE visualization of 130 images in psychophysical experiments. Left: Image feature embedding derived from SimCLR. Right: Embedding derived from participants’ mean property ratings from Experiment 2. In both plots, the dot color represents the most frequently chosen material category for an image in Experiment 3, while the dot size indicates the level of agreement among participants for choosing such a category.

Would simply learning the visual similarity relationship between images be enough to represent materials? To address this question, we used SimCLR^33^, a self-supervised contrastive learning model, as an instantiation of learning an image representation by capturing the visual similarity alone. SimCLR learns an image embedding space of material appearances in a way that it does not presuppose material categories or properties (Fig. 6a, see Methods for detail). We trained SimCLR on our custom dataset of StyleGAN-generated images consisting of prototypical materials and their midpoint morphs. Comparing the resulting model-derived representation with the human perceptual space allows us to probe what visual similarity does and does not account for.

To gain intuition for the learned image representation, we examined embeddings of real photos and synthesized images of materials. First, we assessed the quality of the trained SimCLR model (with ResNet18 as backbone neural network) using real photos with known ground-truth materials. Fig. 6b shows the t-SNE ^34^ embedding of 3000 photos, capturing our curated objects from three material categories (soaps, rocks, and squishy toys) under varying viewpoints and lighting conditions. The embedding formed distinct category clusters, largely aligning with ground-truth labels, indicating that photos with similar visual characteristics tend to come from similar prototypical categories and thus spread in the nearby locations in the learned representation. This suggests that self-supervised learning can yield image representations that capture visual characteristics useful for distinguishing prototypical materials.

Next, we evaluated the relationship between the model’s embedding and human perceptual representation of prototypical and ambiguous materials spanned by the morph continua. Fig. 6c compares the t-SNE embedding of the SimCLR image feature representation of the 130 images tested in Experiments 2 and 3 and that derived from the five human material property ratings. In both plots, the data points were colored by the most frequently chosen material category. There is a lack of correspondence between the SimCLR and perceptual embeddings. Perceptual embedding showed distinct local clusters, mostly separating the perceived rocks and crystals from other materials. In contrast, the model feature embedding exhibited weaker clustering and did not show clear category-based clustering, instead grouping images along their morph continua (see Fig. S7). These findings reveal a gap between human perception and image-similarity–based representations: a material representation learned solely from visual similarities does not fully align with the human perceptual space. Human material representation might not only rely on image-level similarity, but also on an understanding of the semantic properties of materials.

An important characteristic of contrastive learning models is that the learned representation is intrinsically related to the images they encounter during training. One possibility is that this misalignment arises simply because the model was trained on a relatively narrow dataset of material images; a model trained on more diverse content might align better with human perceptual space. To test this possibility, we examined a SimCLR (with ResNet50 as backbone) pretrained on ImageNet, a large-scale dataset encompassing a wide range of objects and material appearances. Prior work has shown that object category information can emerge implicitly from the local similarity structure of SimCLR embeddings^35–37^. The ImageNet-pretrained SimCLR yielded embeddings comparable to those trained on our customized dataset and was similarly able to disentangle the three ground-truth material categories (Fig. S8). However, when we examined the representations of the 130 cross-material morph images, the misalignment remained: the embeddings lacked the clear clustering structure observed in human perceptual space. Thus, although training on a broader range of natural scenes enables the model to capture category-relevant visual features, it does not appear to recover the semantic properties that humans associate with material categories (e.g., rocks are typically hard, whereas squishy toys are soft).

### Discussion

We characterized how humans perceive materials from a continuous range of prototypical and ambiguous appearances of cross-material morphs. Despite large appearance variability, participants can recognize familiar material categories from continuous stimulus space, suggesting they learn the characteristic appearances of each category. Computational analysis using self-supervised deep learning reveals a gap between human and image-similarity-based representations of materials. Our results suggest that the human material representation is supported by the semantic understanding of material properties (e.g., rigidity, smoothness, translucency), which anchors the perceived materials within a meaningful representational space despite broad variation in appearance.

#### Prototypical features for material categorization

What are the visual features that allow for recognizing prototypical materials? One possibility is that each material category is defined by a distinct combination of visual characteristics that distinguishes it from other categories. To investigate this, we analyzed the image generation processes of prototypical materials in StyleGAN generators. Specifically, we examined the rate of change in convolutional weights across different layers of the generator. The generative process for prototypical rocks diverged significantly from that of soaps and squishy toys at the 32 *×* 32 resolution layers, which correspond to image features at mid-to-low spatial scales (Fig. S2). These layers may effectively encode “prototypical features” of rocks, such as object shape and surface texture, that contribute to their unique appearance.

**Fig. 7:**
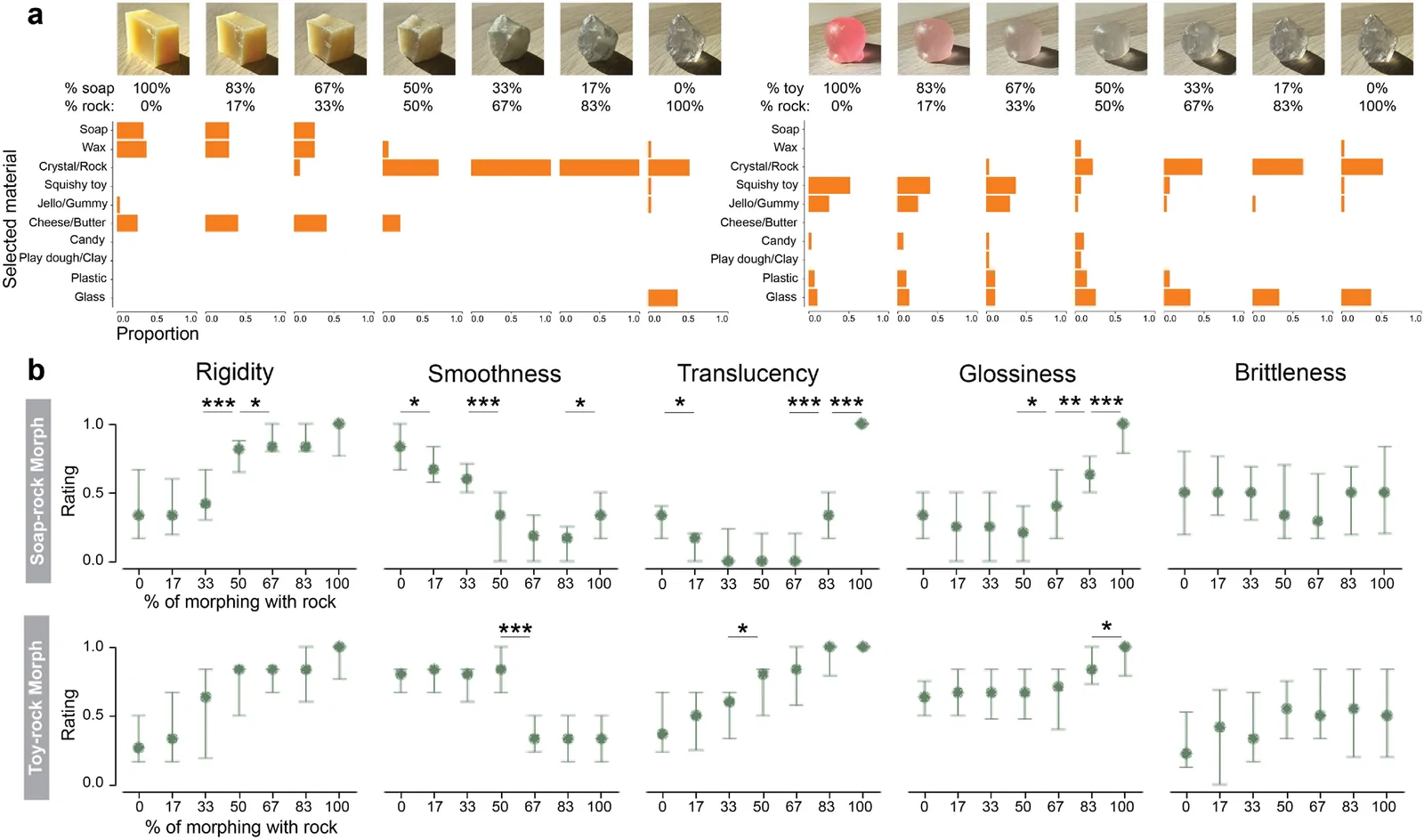
Relationship between material categorization and property rating. (**a**) Distribution of participants’ material category judgments along a soap-rock-toy morph continuum. The images at the top correspond to each morph level. Bar chart indicates the proportion of participants selecting each material category. Left: Soap-rock continuum; Right: Toy-rock continuum; (**b**) Image-wise material property ratings. In each plot, the dot indicates the median ratings, and error bars represent the interquartile range (first and third quartiles) across participants. The top row corresponds to the soap-rock continuum, and the bottom row to the toy-rock continuum. A Kruskal–Wallis test compares property rating distributions between every pair of adjacent morph levels. Significance: “*” *p < 0.05*, “**” indicates *p < 0.01*, and “***” indicates *p < 0.001*.

Sensitivity to these mid-to-low spatial scale image features may facilitate rapid and reliable recognition of rocks. Fig. 7a illustrates participants’ material category judgments along a soap-rock-toy continuum. Notably, adjacent images near the morph midpoint were perceived as different categories. For instance, as the image morphed from 67% Soap-33% Rock to 50% Soap-50% Rock, the categorization judgment abruptly shifted from a mix of “Soap”, “Wax” or “Cheese/Butter” to predominantly “Crystal/Rock”. This categorical shift coincided with a significant increase in perceived rigidity (*p < 0.001*) and a decrease in surface smoothness *(p < 0.001)* (Fig. 7b). Together, the coordinated image feature changes may alter categorization decisions. In other cases, participants remained highly uncertain about the material categories despite the presence of rock-like features (Fig. S5). Understanding how humans learn the association between prototypical visual features and categories through experience, and how such features influence subjective understanding of materials, is a promising direction for future work.

#### Role of semantic properties in organizing categories

Our results suggest that semantic properties play a central role in the mental representation of materials. Specifically, we found that variations in human categorization may not be explained solely by visual similarity (as revealed by MLDS), and that the embedding of materials in a self-supervised contrastive-learning network did not cluster materials the same way humans do. Thus, beyond visual similarity, humans may organize material appearance along more abstract or multisensory dimensions^32,38^. This view is consistent with a growing body of work showing that perceived material properties can capture an interpretable, low-dimensional representational space across very different stimulus media, including photographs, physics-based renderings, paintings, and AI-generated images of materials^18,19,39–41^.

Using object-shaped cross-material morphs, we showed an intricate connection between perceived material properties and categories. In our study, rigidity and surface smoothness emerged as the most effective dimensions for distinguishing among our stimuli (Fig. 5). This link between visual appearance, mechanical properties, and category is presumably learned through interaction with materials^13,42,43^, allowing participants to group materials despite the variations in their optical properties. Optical properties, on the other hand, appear to play a more nuanced role in categorization than previously reported. While previous studies observed that optical properties, such as glossiness, strongly predict material categories^21,23^, we found relatively weak correlations between these properties (i.e., glossiness, translucency) and categorization. This discrepancy may be attributed to our stimulus design, which allowed for concurrent variation in appearances and multiple material properties. When object shape is fixed, optical cues can dominate category judgments. However, when optical cues are shared across categories, they become less effective for categorical distinction and instead contribute to more refined, within-category differentiation (Fig. 5). Together, these findings suggest that different material properties contribute in distinct ways to the mental representation of materials.

#### Representation of perceptual continua and categories

Whether the brain represents visual content as discrete categories or continuous dimensions remains widely debated^3^. Morphing offers a means of creating controlled, continuous variation in stimuli that reflects changes in perceptual dimensions and categories. In particular, cross-category morphs provide a foundation for studying categorical perception—a phenomenon reported in auditory (e.g., phoneme perception^1,44^) and visual domains (e.g., color^45^ and face^5^ perception). This phenomenon has motivated investigations into whether and how neural activity reflects category membership in object and shape^46,47^. Beyond this category-centric view, visual representations can alternatively be characterized by continuous dimensions that capture the underlying conceptual and perceptual properties of stimuli. These dimensions have been shown to predict human categorization and similarity judgments of objects and materials^3,32,48^. Increasing evidence suggests that the brain may integrate both representational formats. For example, the study of facial expression revealed that both continuous and categorical representations are presented in the brain’s face-selective regions^5^. By examining the brain’s spatiotemporal dynamics in processing vocal emotion, recent work proposes a reconciliation of these two opposing views, suggesting that emotion perception involves the interplay of cerebral networks with different representational dynamics that emphasize either categories or dimensions^49^.

Our cross-material morphs provide an opportunity to examine how the brain balances category-based and dimension-driven representations. On the one hand, recognizing and categorizing materials requires organizing a potentially infinite range of appearances into finite sets of semantically meaningful classes. The abrupt shifts in category judgments observed near the morphing midpoint suggest that materials may, at least in part, be represented categorically. Future work could further test categorical perception of materials—for instance, whether perceptual discrimination of materials is enhanced across category boundaries compared to within categories. On the other hand, materials can be represented in terms of different semantic properties. We can often judge properties such as glossiness without explicitly identifying the material, whereas properties like edibility or softness may require more explicit material recognition. Thus, understanding material perception may require considering the observer’s perceptual goals^50,51^. For example, when planning footsteps on an icy road, variation in surface glossiness may become increasingly critical. In another context, when evaluating whether an object is edible, categorically distinguishing between candy and crystal becomes more relevant. Future research could investigate how the behavioral and neural representations of materials are shaped by different perceptual goals and task demands.

#### Limitations

In this study, we focused on a limited set of prototypical material categories. Expanding the stimulus space to include additional materials, such as candy, wood, or metal, would increase the diversity of visual appearances and broaden the range of material properties available to participants. Because our stimuli were created by morphing between materials that differed substantially in rigidity, translucency, and surface smoothness, these properties naturally emerged as the dominant perceptual dimensions. A more diverse material set would likely reveal a richer, more nuanced structure of material representation, in which additional properties become more important, and the relationship between material categories and properties becomes more complex. Even so, we expect morphing between distinctive prototypical materials would yield a categorization tuning curve similar to the one we observed. Material representation would continue to be organized by similarities across multiple properties, though the relative importance of different properties may shift with the characteristics of the stimulus set. Beyond the properties measured here, other multisensory properties (e.g., haptic, auditory) and affordances (e.g., edibility) could emerge as behaviorally relevant dimensions that further structure material representation.

We also acknowledge that our stimuli feature materials against simple backgrounds with little variation. In natural settings, however, materials typically appear in richer and more diverse contexts, and certain materials are often coupled with particular environments (e.g., soaps in bathrooms, rocks in outdoor landscapes). Such contextual information can facilitate object recognition, especially when the object itself is ambiguous^52,53^, and it is plausible that similar context-dependent effects shape material perception. Our study characterizes material categorization and property estimation under minimally informative background scenes, and therefore does not directly address how natural scene context contributes to material perception. Future work could systematically vary background scenes — from neutral backgrounds to semantically congruent and incongruent ones — to probe the influence of context on material reasoning, particularly for ambiguous materials that blend features across categories.

### Conclusion

Our findings show that humans, through experience, learned to associate specific diagnostic visual features with material categories. Meanwhile, learning meaningful properties is crucial to structuring the mental representation of materials, within and across categories. More broadly, our work provides a promising avenue towards investigating how the brain represents semantically rich stimuli using AI-generated photorealistic morphs.

## Methods

### Stimulus creation

All images used in the experiments were generated by StyleGAN2-ADA^54^, a generative deep learning model trained on our curated image datasets. Specifically, we first trained StyleGAN on a Soap Dataset (8085 photos), and then separately fine-tuned it on the Rock Dataset (3180 photos) and Squishy Toy Dataset (1900 photos). Upon training, we obtained three distinct models: soap ($w_{soap}$ and $G_{soap}$), rock ($w_{rock}$ and $G_{rock}$), and squishy toy ($w_{toy}$ and $G_{toy}$) models. Each model comprises two components: a latent code (*w ∈ W*) and a generator (*G*). As a note, only the weights of the generator are learnable parameters. The latent code, a tensor in dimension of 18 *×* 512, is drawn from the latent space (*W*) resulted from the StyleGAN training (i.e., $w_{soap} \in W_{soap}$, $w_{rock} \in W_{rock}$, and $w_{toy} \in W_{toy}$). With these trained models, we can synthesize an image of the prototypical material (100% Soap, 100% Rock, or 100% Squishy toy) from a corresponding pair of latent code and generator. Specifically, each of the 18 layer-wise latent vectors in $w_{soap}$ is injected into the convolutional layers of $G_{\text {soap}}$ at matching spatial resolutions (from 4 × 4 to 1024 × 1024 pixels) to generate a soap image. The same procedure was applied to the rock and squishy toy models.

Given a pair of source and target materials, we can create a morph continuum by applying linear interpolation between the corresponding models, including both their latent codes (across all 18 layers) and the generator’s convolutional weights:

1 $$\begin{aligned} w_{\lambda } = w_{source} + \lambda (w_{target} - w_{source}) \end{aligned}$$

2 $$\begin{aligned} G_{\lambda } = G_{source} + \lambda (G_{target} - G_{source}) \end{aligned}$$

where $w_{source}$ and $w_{target}$ are latent codes of the source and target materials, and $G_{source}$ and $G_{target}$ are the corresponding generators. The value of *λ* indicates the interpolation step. Hence, inserting the $w_{\lambda }$ (in dimension of 18 *×* 512) into the interpolated $G_{\lambda }$ generates an image of morphed material.

We preselected thirty unique StyleGAN-generated images, ten from each prototypical material category of soap, rock, and squishy toy (see Fig. S1 Leftmost, Middle, and Rightmost columns). We then created unique morph continua by transforming the soap into the rock and the squishy toy into the (same) rock, each with five intermediate morph levels. The value of $\lambda \in [0.17, 0.33, 0.5, 0.67, 0.83]$ controls the “percentage of rock” in the resulting morphed image. For example, *λ = 0.5* generates a 50% Soap-50% Rock image, representing the combination of 50% of yellow soap and 50% of transparent rock (second image from the left in Fig. 1c).

Applying this interpolation process on different soap-rock and toy-rock pairs leads to 130 images, encompassing 20 seven-level morph continua: 10 from soap-rock morph and 10 from toy-rock morph. A soap-rock morph continuum included: 100% Soap, 83% Soap-17% Rock, 67% Soap-33% Rock, 50% Soap-50% Rock, 33% Soap-67% Rock, 17% Soap-83% Rock, and 100% Rock; a toy-rock morph continuum followed a similar structure. In total, we had 13 unique morph levels. As shown in Fig. S1, the generated materials cover two major lighting environments: strong indoor lighting (rows 1 to 5), and overcast indoor lighting (rows 6 to 10). Stimuli for all subsequent experiments were from this dataset of 130 images.

## Experiment 1: Visual similarity judgment

### Participants

We recruited a group of 20 participants (9 females, mean age = 22.10, range 18–27). The sample size was chosen on the basis of similar behavioral studies using MLDS to measure stable estimation of perceptual scale^24,55–57^. All participants were undergraduate or graduate students at American University with normal or corrected-to-normal vision, and all collected data were included in the analyses. Participants were paid $20 per hour in the form of a giftcard for their participation.

#### Ethics approval

The study was approved by the Institutional Review Board at American University. All experiments were performed in accordance with the relevant guidelines and regulations. The study was conducted following the rules of the Declaration of Helsinki.

#### Informed consent

Informed consent was obtained from all participants.

### Procedure

MLDS triads were independently created from seven-level soap-rock morphs and toy-rock morphs. For each seven-level morph continuum, we fully sampled 35 unique triads (i.e., distinct combinations of 3 out of 7 images) (Fig. 2a). On each trial, participants judged which image pair in the triad was more similar in visual appearance and responded by pressing "1" for the left pair or "0" for the right pair.

Participants completed 20 blocks of MLDS judgments: 10 for soap-rock and 10 for toy-rock morph continua, in a dimly lighted laboratory (Fig. S1). Each block contained 35 triads from a single morph continuum. Within each block, the 35 triads were randomly ordered and repeated twice (i.e., 70 trials per block). The 20 blocks were presented in a different randomized order for each participant. Participants viewed the stimuli at a distance of 50 cm from the screen. Each image in a triad spanned approximately 9.14 degrees in spatial extent. The leftmost and rightmost images were approximately separated by 24.27 degrees, with each image located at the same distance from the center image.

Stimuli were displayed via an Apple iMac computer with a 21.5-inch Retina Display, with a resolution of 1920 pixels *×* 1080 pixels. PsychoPy v.2021.1.2 was used to present the stimuli and collect the data.

## Experiments 2 & 3: Material property rating and material categorization

### Participants

A new group of 27 healthy adults (13 females, mean age = 22.07, range 18–27) participated in both Experiments 2 and 3. All participants gave informed consent in accordance with the procedures approved by the institutional review board. Before we recruit the subjects, a power analysis for t-tests of means (calculated using the *pwr* package in the R environment) indicated that a sample of 26 participants would be needed to obtain statistical power at 0.80 (significance level *α = 0.05*) to detect a medium effect difference of material property ratings and material categorization confidence ratings among morph levels. To evaluate the power of material category selection, we computed the proportion power at 0.80 (significance level *α = 0.05*) and found an effect size of 0.76 with 27 participants. The participants were undergraduate and graduate students from university, and all had normal or corrected-to-normal vision. Participants were paid $20 per hour in the form of a giftcard for their participation. No participants were excluded from the data analysis.

#### Ethics approval

The study was approved by the Institutional Review Board at American University. All experiments were performed in accordance with the relevant guidelines and regulations. The study was conducted following the rules of the Declaration of Helsinki.

#### Informed consent

Informed consent was obtained from all participants.

### Experiment 2: Material Property Rating

Participants were presented with the full dataset of 130 images in the same pre-randomized order. In each trial, they judged five material properties of an image on a seven-point scale (Fig. 2b). The image remained on the screen until the participant rated all properties. These material properties are defined as follows:

- Translucency: to what extent does the material appear translucent?
- Glossiness: to what extent does the material appear glossy or shiny?
- Smoothness: to what extent does the material’s surface feel smooth when touched?
- Rigidity: to what extent does the material appear hard, rigid, or stiff?
- Brittleness: to what extent does the material appear brittle?

Before the main experiment began, participants completed a practice block of 16 trials. Practice-trial stimuli were drawn from the 130-image stimulus set and sampled to cover all five categories (soap, soap-rock morph, rock, toy-rock morph, and squishy toy), ensuring that participants saw a diverse range of material appearances before providing ratings for analysis. Each participant judged each image only once, except that images presented during the practice block were judged again in the main experiment. Data from the practice trials were not included in any analyses.

### Experiment 3: Material Categorization

Participants judged the material category of the displayed image by selecting from ten candidate material categories: soap, crystal/rock, squishy toy, wax, jello/gummy, plastic, glass, cheese/butter, candy, and play dough/clay (Fig. 2c). Participants were instructed to “select the material that best describes the object in the image.” The candidate categories were based on the material naming results from our previous study, in which participants verbally described materials in the StyleGAN-generated images, similar to those used in the current study^22^. Hence, participants rated their confidence in material category judgment with a five-point scale. The image remained on the screen until the participant selected the material category and assigned the confidence rating. Participants were presented with the same set of 130 images as in Experiment 2, but in a different pre-randomized order. Before the main experiment, participants completed a practice block using the same 16 images as in the practice block of Experiment 2. Data from the practice block was excluded from subsequent analyses.

### Procedure

Participants viewed image stimuli at a distance of 77 cm from the screen, in a dimly lighted laboratory. Each image subtended approximately 9.35 degrees of visual angle. The stimuli were displayed using the same device as in Experiments 1. Participants sequentially completed Experiment 2 (Material Property Rating) and 3 ( Material Categorization) in two separate sessions. Experiment 2 and 3 spanned approximately 1.5 hours and 1 hour, respectively. Material Property Rating was completed before Material Categorization to minimize the potential bias of our provided candidate material categories on participants’ judgment of material properties.

## Self-supervised learning SimCLR

Self-supervised learning enables deep convolutional neural networks (DCNNs) to capture meaningful visual representations without relying on labeled image data^35,36^. Here, we trained a DCNN with SimCLR^33^, a contrastive self-supervised learning framework that enables the DCNN to learn the intrinsic relationships among image samples. During training, each image is augmented with random transformations to create different views of the same instance (see Fig. 6a). These augmented views are processed through a DCNN backbone to obtain a 512-dimensional image embedding, which is then mapped to a fully connected lower-dimensional space (128 dimensions) via a multilayer perceptron for contrastive loss optimization. Contrastive learning encourages the model to maximize agreement between differently augmented views of the same data example, while also separating them from the embedding of other images. In other words, the contrastive objective causes representations of different views of the same image exemplar to attract one another in the embedding space, while causing representations of views from different exemplars to repel one another. Upon SimCLR training, the DCNN backbone can be used to extract image embeddings (512 dimensions) for novel image samples.

### Training dataset and data augmentation

The training dataset comprised 10,800 StyleGAN-generated images. It included three prototypical materials (i.e., 100% Soap, 100% Rock, and 100% Squishy toy) and three morph midpoint materials (i.e., 50% Soap-50% Rock, 50% Squishy toy-50% Rock, and 50% Soap -50% Squishy toy), with each category containing 1,800 images. All images were resized to 224 pixels *×* 224 pixels. During training, data augmentation was applied to create varied views of each sample: random resized crop, random horizontal flip, color jitter, random grayscale, gaussian blur, and ImageNet normalization.

### Training details

We used a standard ResNet18^58^ as the DCNN backbone in SimCLR. The SimCLR was trained for 200 epochs with a batch size of 512 images using stochastic gradient descent, with momentum = 0.9 and weight decay = 5e-4. The learning rate, beginning at 0.06, was dynamically adjusted via a Cosine Annealing learning rate scheduler. The training was implemented using Lightly (https://github.com/lightly-ai/lightly), a Pytorch implementation of SimCLR. We then used the trained ResNet18 backbone to derive image embeddings for further analysis. Our model was trained on a single NVIDIA RTX 4090 GPU.

## Supplementary Information

Supplementary Information.

## Data Availability

The image datasets used to train the generative artificial neural networks (StyleGAN) are publicly available on the Figshare repository (https://figshare.com/articles/dataset/Space_of_Morphable_Material_Appearance_Datasets_and_Models/27038518). Experimental stimuli, human data, and data analysis code are publicly available on the OSF repository (https://osf.io/zgcxn/?view_only=f9e96b319cd349a4bead96bd4454f3af).
