# Supplementary material for: Material recognition from cross-category morphing
Source: Sci Rep. 2026 Oct 1;16:30508. doi: 10.1038/s41598-026-59920-x (PMC13630578; doi:10.1038/s41598-026-59920-x)
Supplement: Supplementary file 1 [file 41598_2026_59920_MOESM1_ESM.pdf]

Supplementary Information

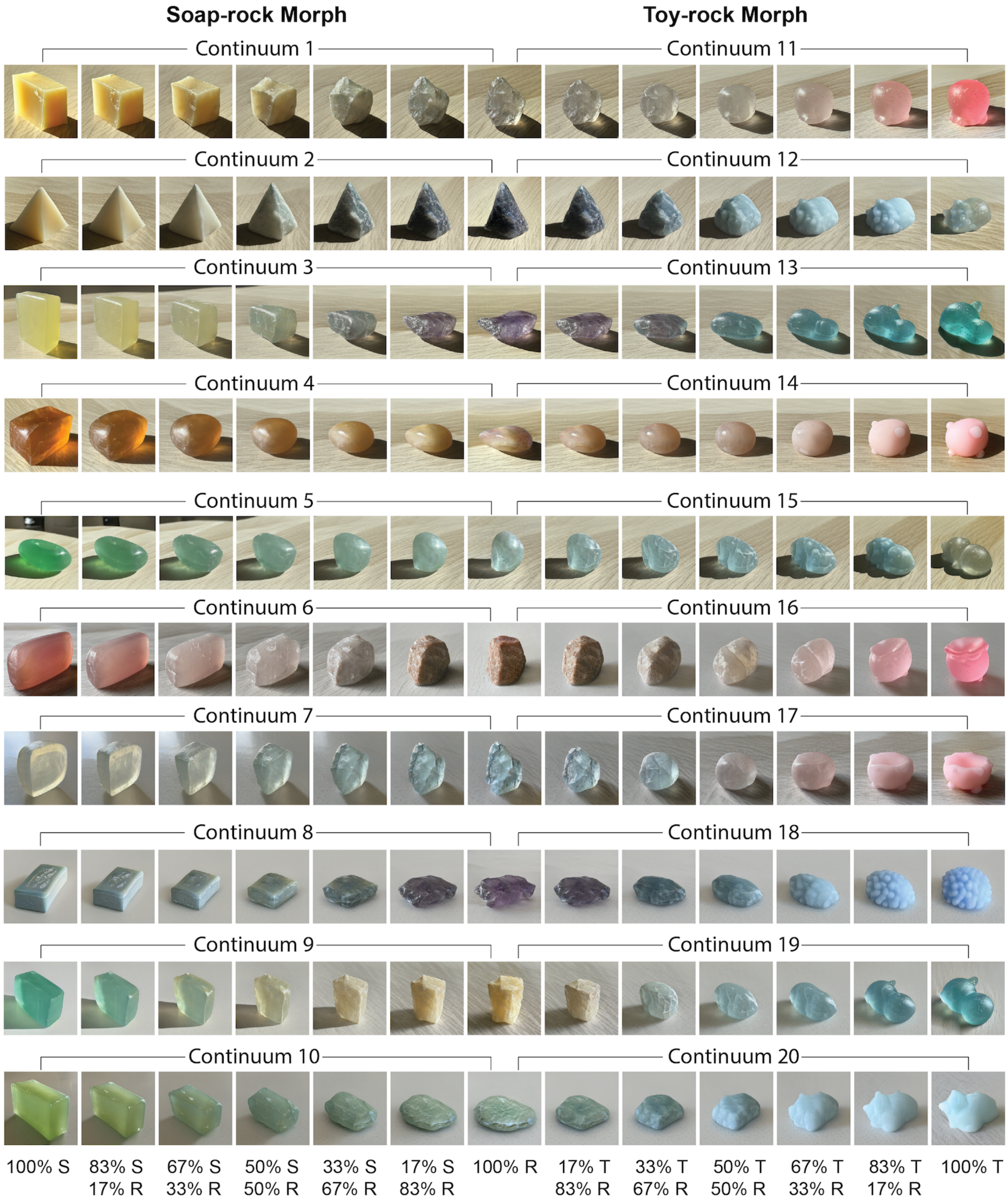

Figure S1. Image dataset for stimulus sampling. Each row presents a thirteen-level morph continuum transitioning between soap, rock, and squishy toy. These 130 images served as stimuli for all three psychophysical experiments. For Visual Similarity Judgment (Experiment 1), MLDS triads were independently created from 20 seven-level morph continua—10 for the soap-rock morph continuum and 10 for the toy-rock morph continuum. For Material Property Rating (Experiment 2) and Material Categorization (Experiment 3), participants judged the full set of 130 images.

Comparison of StyleGAN generators of the prototypical materials

We analyzed the rate of change of the models' convolutional weights between a pair of material generators. As a note, we used the pretrained soap generator (G_{soap}) as the starting point, separately applying end-to-end fine-tuning to obtain the rock (G_{rock}) and squishy toy (G_{toy}) generators. The generator starts from a learned constant input of size 4 × 4 × 512 and gradually expands the spatial resolution via affine transformation layers. At each resolution, from 4 pixels × 4 pixels to 1024 pixels × 1024 pixels, an additional single convolution layer (toRGB) transforms the feature maps into the RGB image.

Given a pair of trained StyleGAN generators G and G', we computed the average change rate of weights at each convolution layer (here, we omit the bias and other parameters in the normalization layers): $\Delta = \frac{1}{N}\sum_{i=1}\frac{|\theta_i' - \theta_i|}{|\theta_i|}$, where N is number of parameters, θ_i and θ_i' is the i-th parameter in generator G and G'. For each pair of prototypical material generators, we computed Δ across layers corresponding to different spatial scales, from 4 pixels $\times$ 4 pixels (i.e., b4.conv and b4.toRGB) to 1024 pixels $\times$ 1024 pixels (i.e., b1024.conv and b1024.toRGB). Larger value indicates bigger difference between a pair of generators at a particular layer.

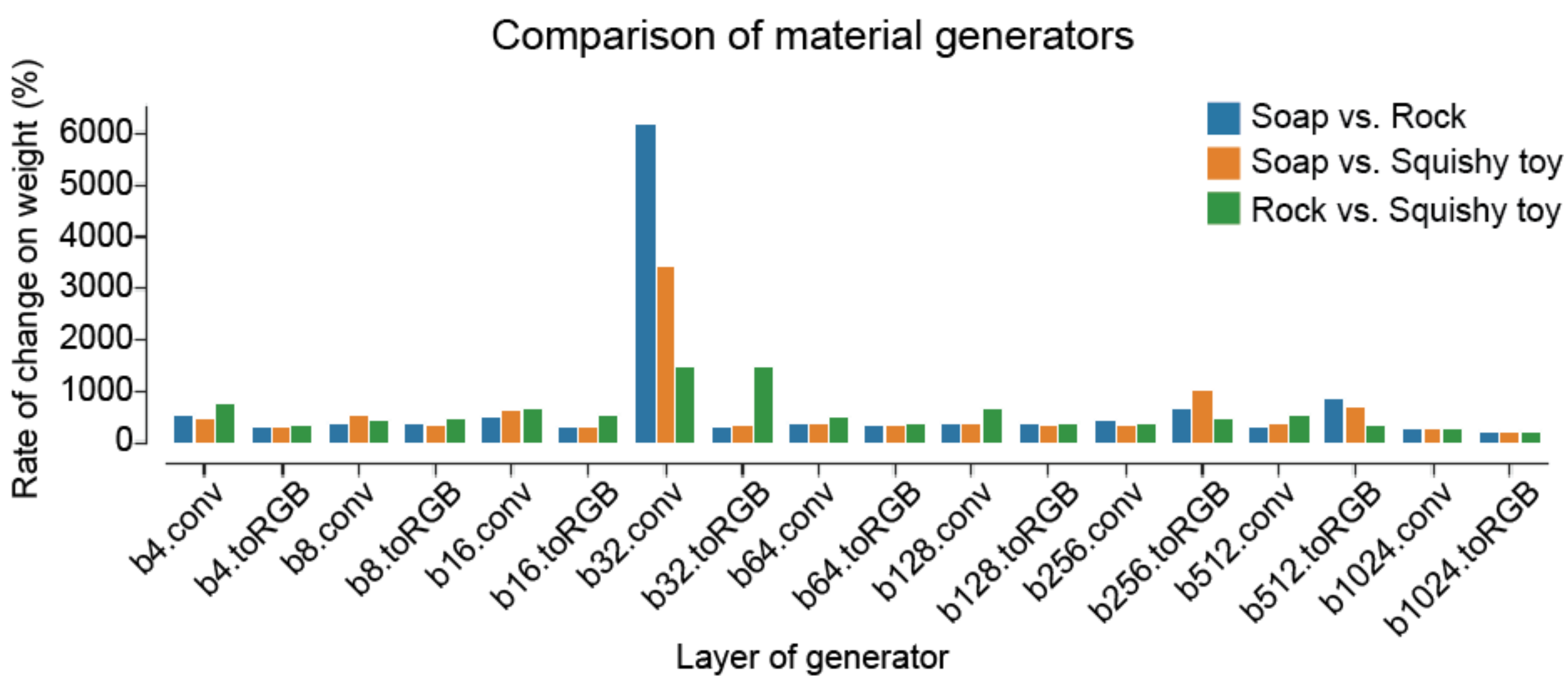

Figure S2. Comparison of StyleGAN generators of the prototypical materials. The plot displays the weight change rate at each convolutional layer between two generators of prototypical materials.

Category selection in Material Categorization experiment

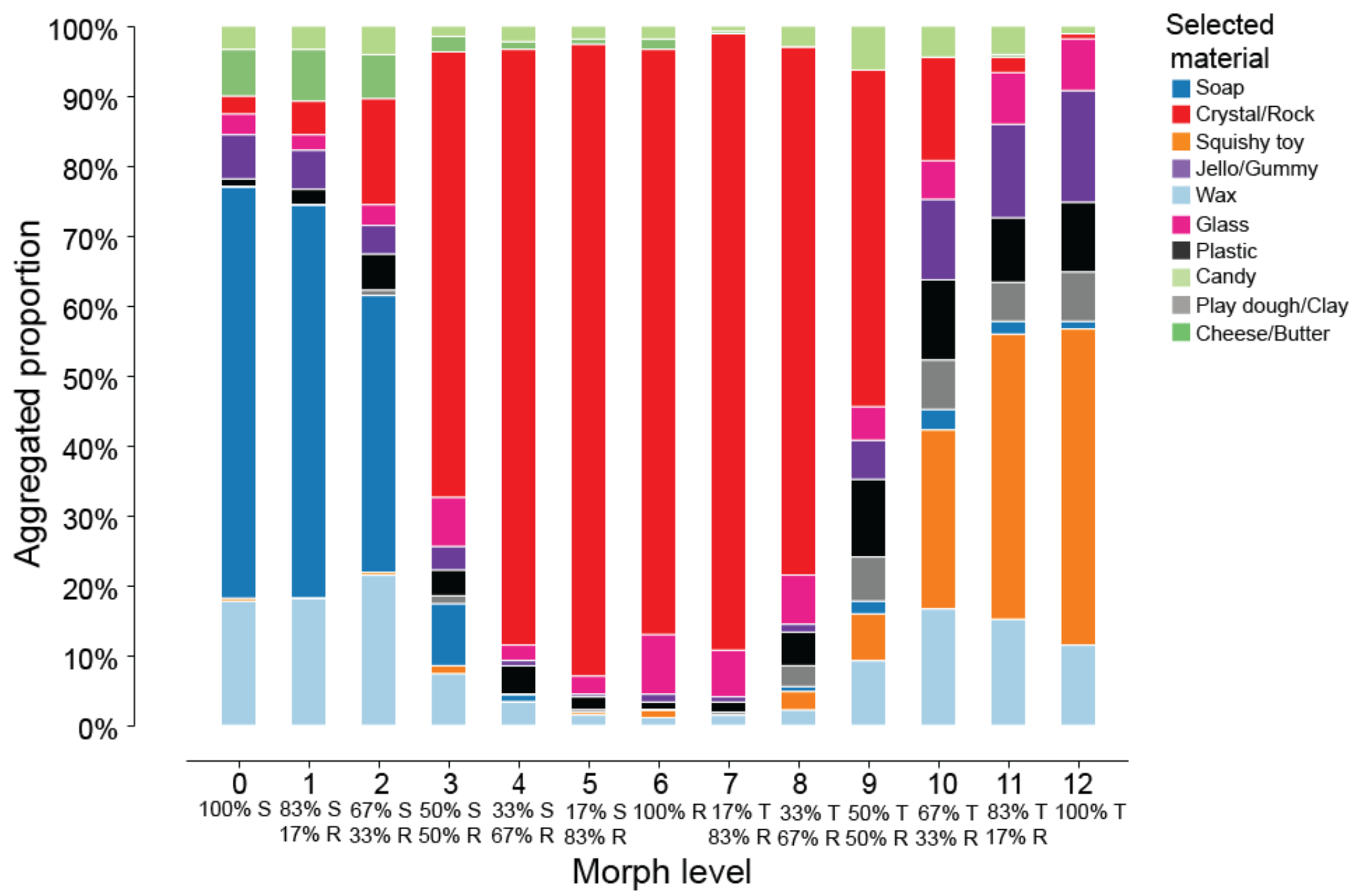

Figure S3. Distribution of material selection across morph levels. The stacked bar chart represents the aggregated proportion of participant selections for each material category at each morph level. 100%S, 100%R and 100%T indicate prototypical soap, rock, and squishy toy, respectively. 50%S-50%R and 50%T-50%R indicate the soap-rock midpoint and squishy toy-rock midpoint, respectively.

Confidence judgment of material categorization

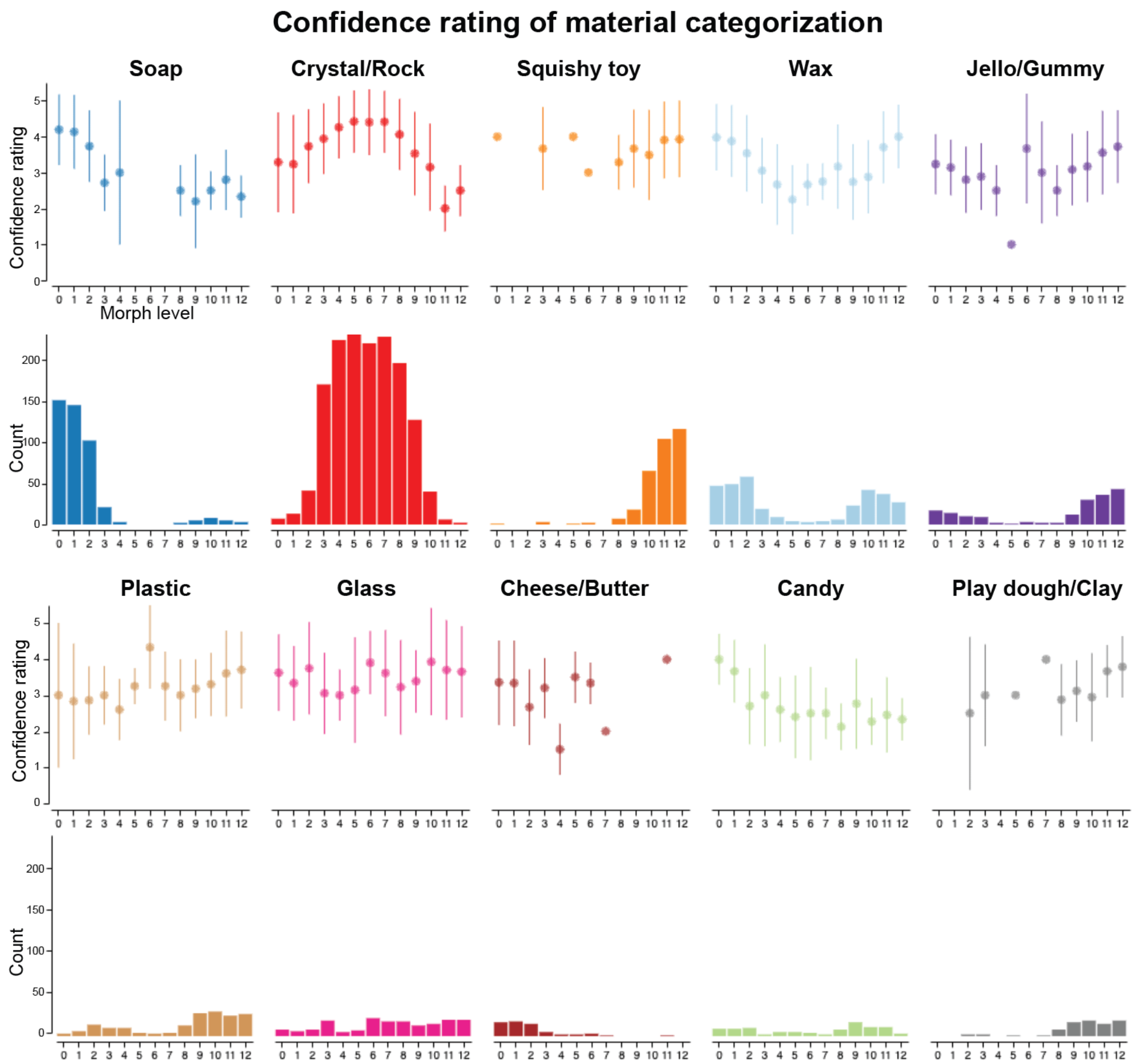

Figure S4. **Confidence rating of material categorization.** Each panel displays results from the Material Categorization experiment based on participants' selected material categories. The dot plot shows mean confidence ratings at each morph level, with error bars representing standard deviation across independent trials. The bar chart shows the distribution of the total number of times participants classified images into the corresponding material category.

Perceptual ambiguity of material categorization

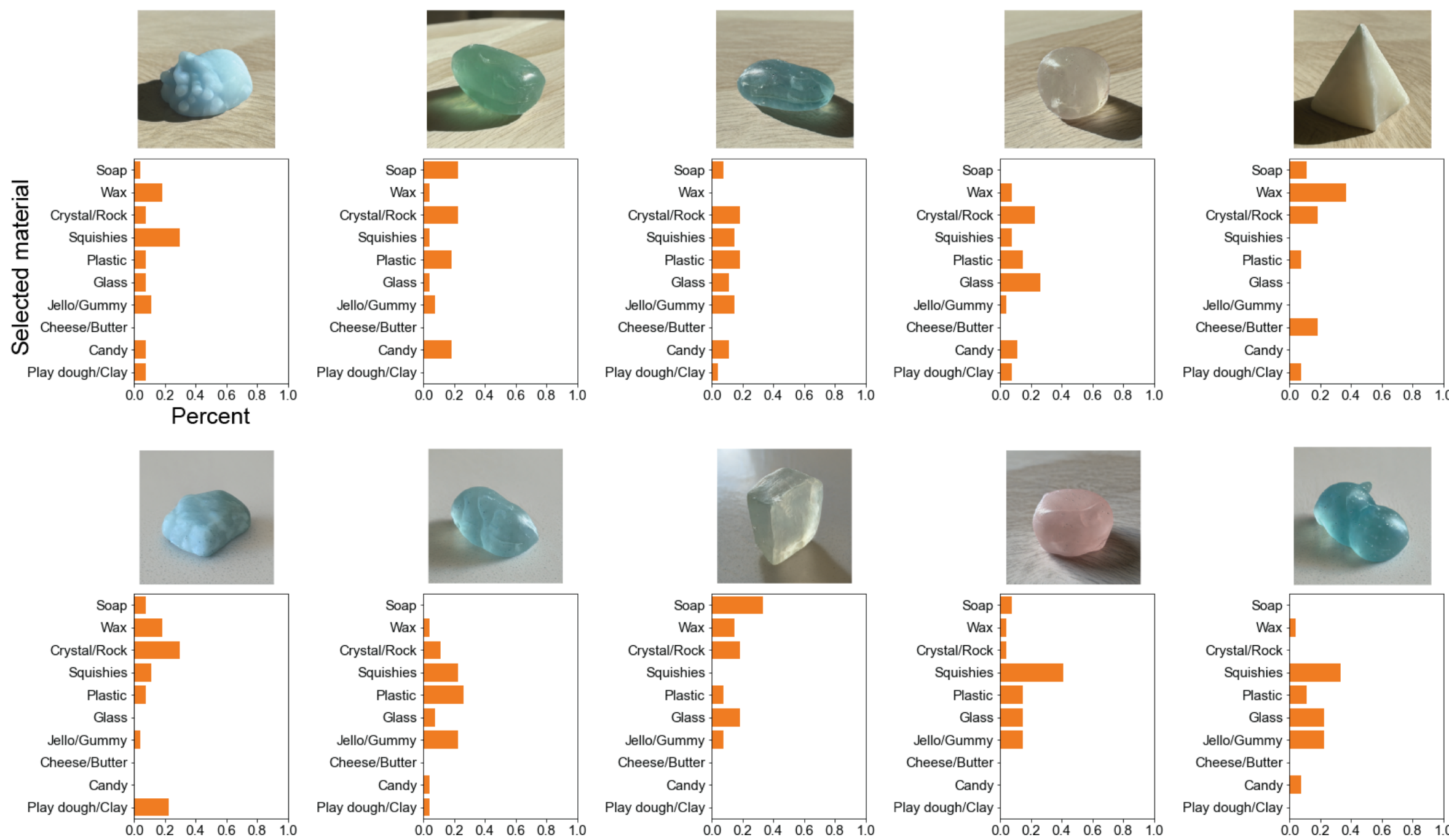

Figure S5. Most ambiguous images in Material Categorization experiment. Bar chart indicates the proportion of participants selecting each material category. These images have the highest entropy of the distribution of selected categories.

Visual similarity judgment via MLDS

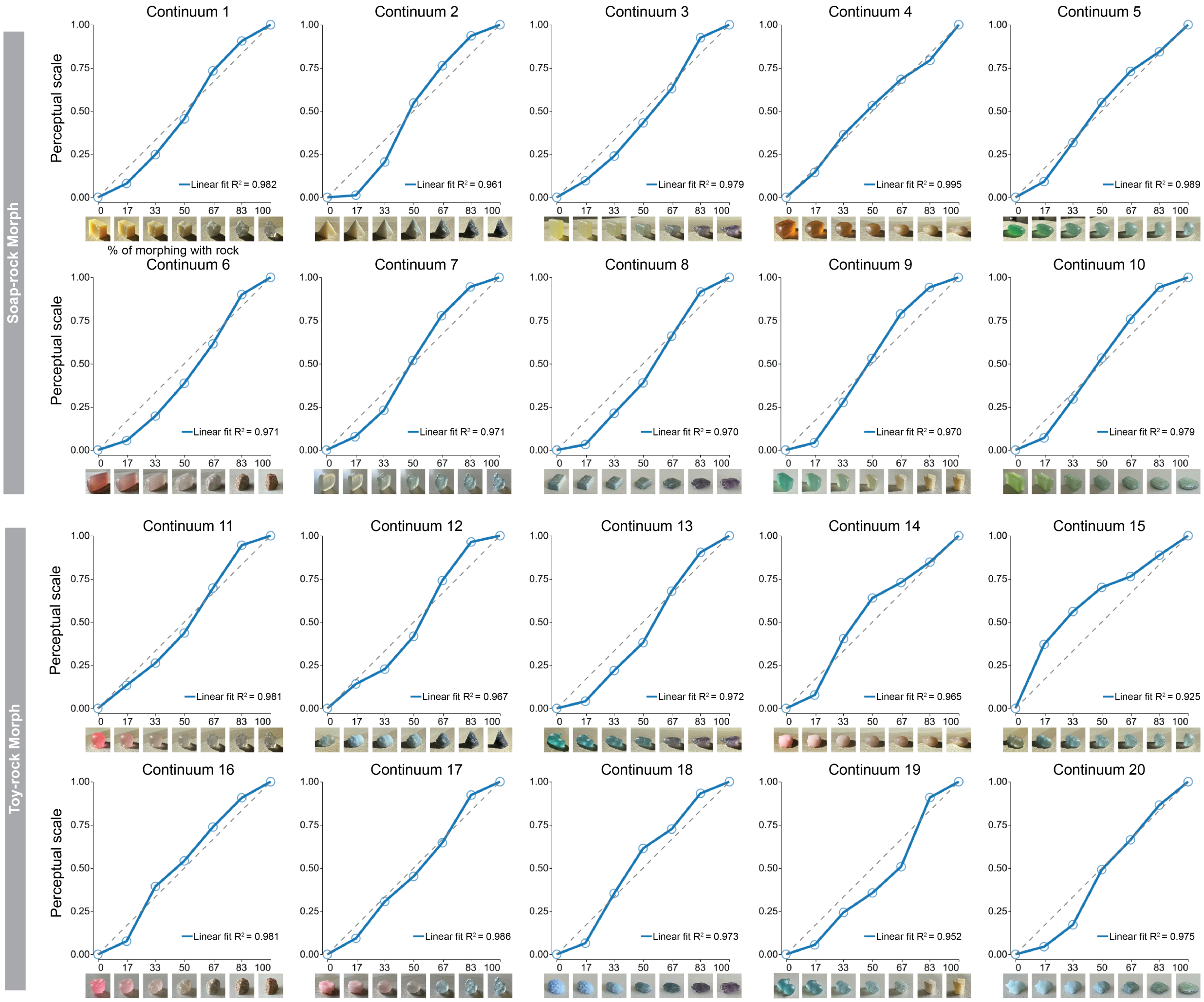

Figure S6. Visual similarity judgment across tested morph continua. In each plot, the blue curve shows the MLDS result, representing the mean perceptual scale for the morph continuum averaged across all participants. The x-axis represents the morph level, with higher values corresponding to a greater proportion of the target rock image in the morph. The gray dotted diagonal line represents the linear variation of perceptual judgments. R-squared value of the linear fit of the MLDS curve is shown in the bottom right corner.

t-SNE embeddings of SimCLR image feature and human material property rating

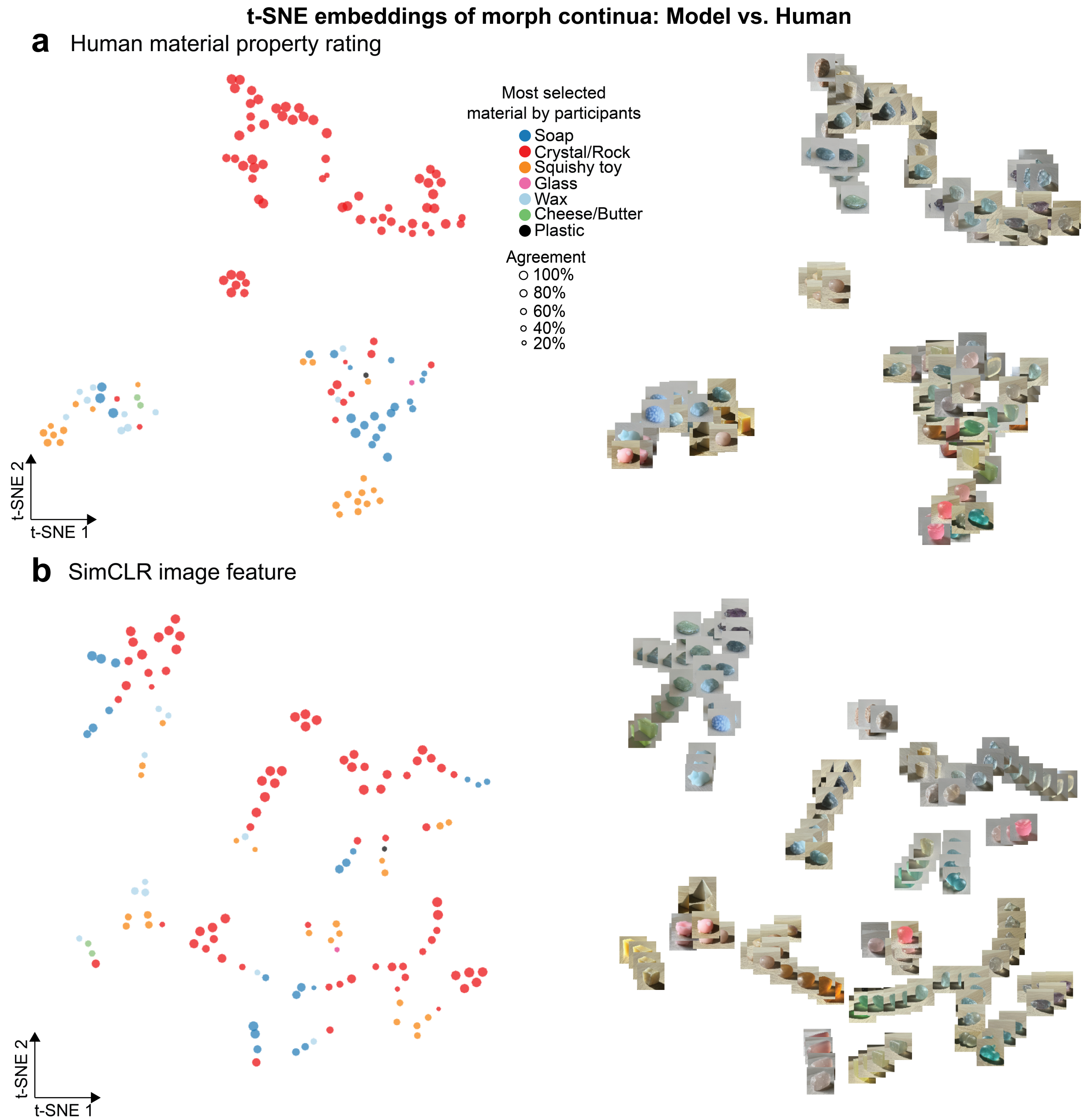

Figure S7. Comparison of material representations derived from human material property ratings and self-supervised deep learning model SimCLR. (a) t-SNE embedding of 130 images from psychophysical experiments, using mean material property ratings from Experiment 2. Left: The dot color represents the most frequently chosen material category for an image, while the dot size indicates the level of agreement among participants for choosing such a category. Right: The same t-SNE annotated by corresponding images. (b) t-SNE embedding of the same 130 images, using image feature representation derived from SimCLR (ResNet18 as backbone neural network). Data points are annotated in the same way as (a).

Comparing SimCLR representations from ImageNet pretraining and customized-dataset training

We tested whether the image representation derived from SimCLR pretrained on ImageNet — a large-scale dataset spanning a diverse range of objects and materials — would better align with the human perceptual space. The pretrained SimCLR uses

ResNet50 as its backbone network and produces a 2048-dimensional feature space. For a fair comparison, we also trained a SimCLR with the same ResNet50 backbone on our customized material image dataset (same dataset used for training the ResNet18 backbone reported in main paper). This model was trained for 200 epochs with a batch size of 128 images, using stochastic gradient descent (momentum = 0.9, weight decay = 5e-4) and a learning rate initialized at 0.06 with Cosine Annealing. Fig S8 shows the embeddings of the 3,000 real material photographs and 130 images in the psychophysical experiments for both models, replotting Fig 6 in the main paper for direct comparison.

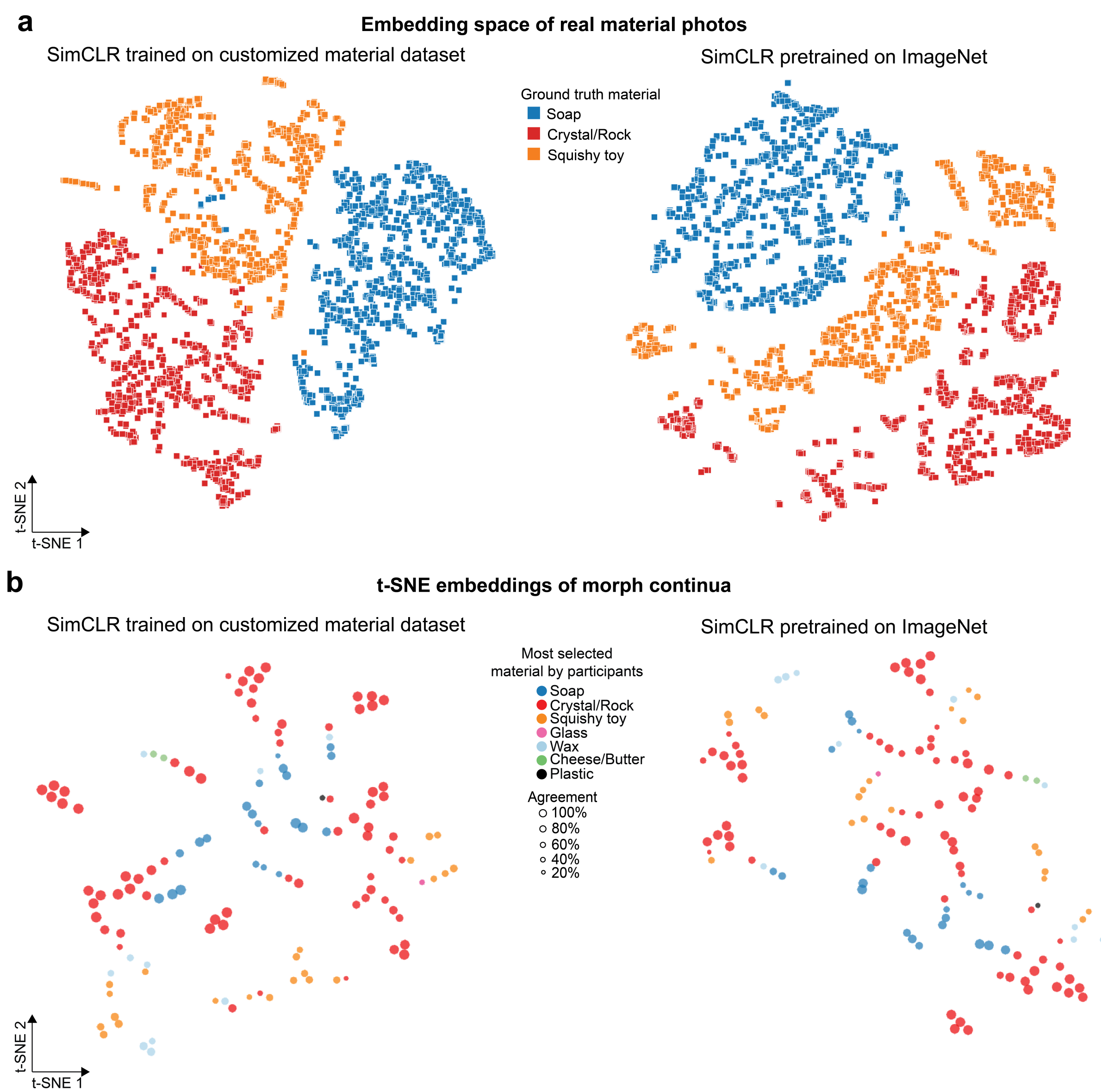

Figure S8. Image embeddings derived from customized and ImageNet-pretrained SimCLR models. (a) t-SNE embeddings of 3,000 photographs of real materials from three categories: soaps, crystals/rocks, and squishy toys. (b) t-SNE embeddings of the 130 images used in the human psychophysical experiments. Both the customized and pretrained SimCLR models use a ResNet50 backbone. The models were trained on different datasets: the customized model was trained on our 10,800 StyleGAN-generated material images, while the pretrained model was trained on ImageNet-1K.

Representational Similarity Analysis

Material categorization and property rating

We constructed the image-by-image Representational Dissimilarity Matrices (RDMs) to depict the representational space of material categorization and that of material property rating (Fig S9). The material categorization RDM captured pairwise dissimilarities between the frequency distributions of material category selections (i.e., the distribution of participants' selection of 10 candidate materials in Experiment 3) across 130 images. In other words, for any pair of stimuli (stimulus A and B), both stimulus A and B were represented by a 10-dimensional (10-D) vector based on the frequency that each of the 10 categories was selected, and then we measured the Euclidean distance between these two 10-D vectors.

We constructed material property RDMs by computing pairwise Euclidean distances between images based on their mean ratings for each property in Experiment 2. The material categorization RDM and the material property RDMs all had dimensions of 130×130. We then conducted the Representational Similarity Analysis (RSA) by computing the Pearson correlation between two RDMs.

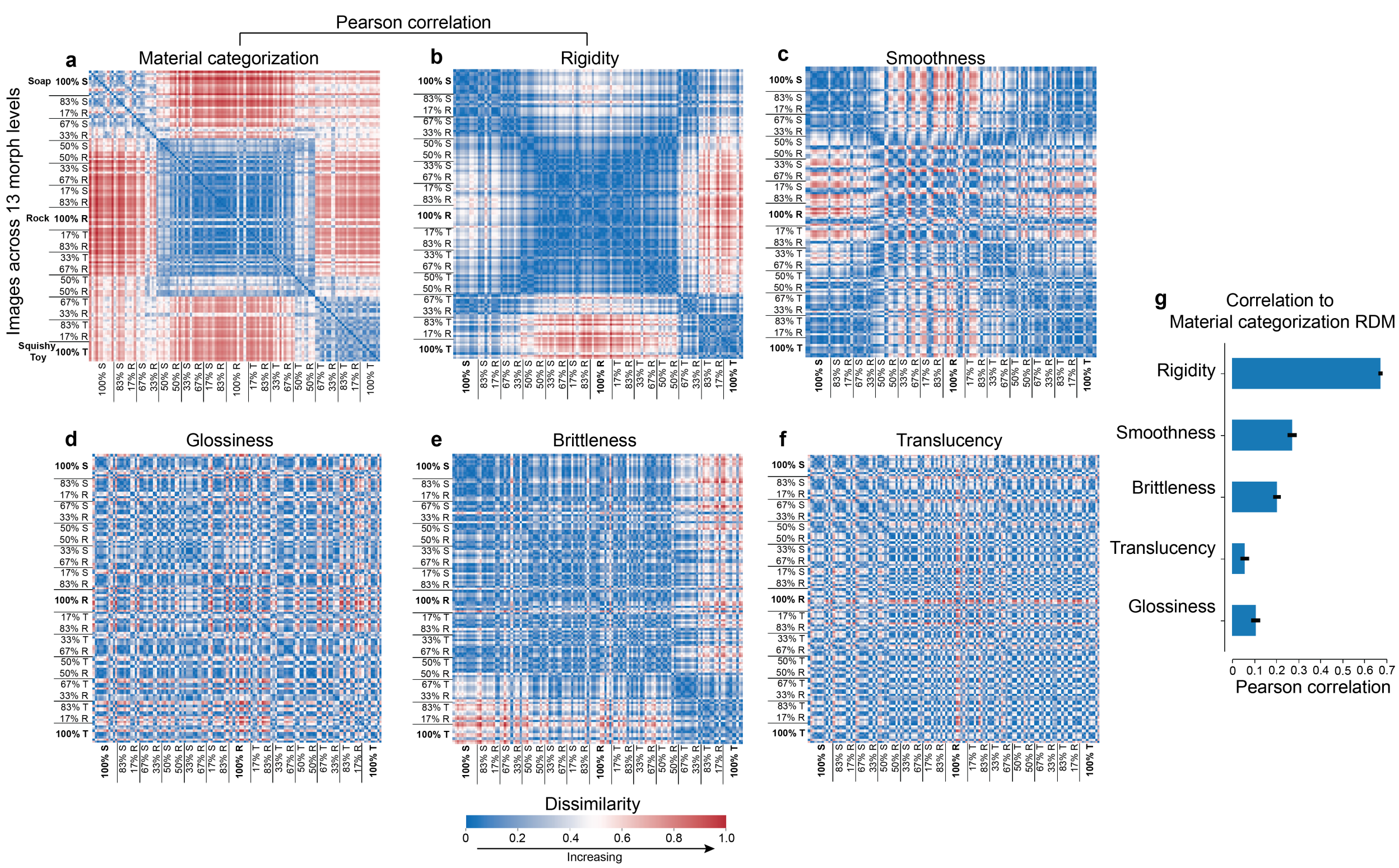

Figure S9. Image-by-image RDMs (a) Material categorization RDM (c-f) Material property RDM based on each of five material properties: rigidity, smoothness, glossiness, brittleness, and translucency. The axis of the RDM represents the 130 images, ordered by the 13 morph levels from soap (100%S), to rock (100%R), and to squishy toy (100%T). Each RDM was normalized to 0 to 1 range. Darker blue cells in the RDM indicate smaller differences between a pair of images, and darker red cells indicate larger differences. (g) Pearson correlation between material categorization RDM and material property RDMs. The blue bar shows the mean correlation, and the error bar indicates the 95% confidence interval via bootstrapping (1000 resampling).

SimCLR image representation and human perception

We also applied RSA to quantify the alignment between SimCLR-derived image representations and human perception (Fig S10). The model RDM was constructed from pairwise Euclidean distances between the feature vectors of the 130 images in the psychophysical experiments. The human perception RDM is based on pairwise Euclidean distances between five-dimensional vectors of mean material property ratings (i.e., each image is represented by five properties: rigidity, smoothness, translucency, glossiness, and brittleness).

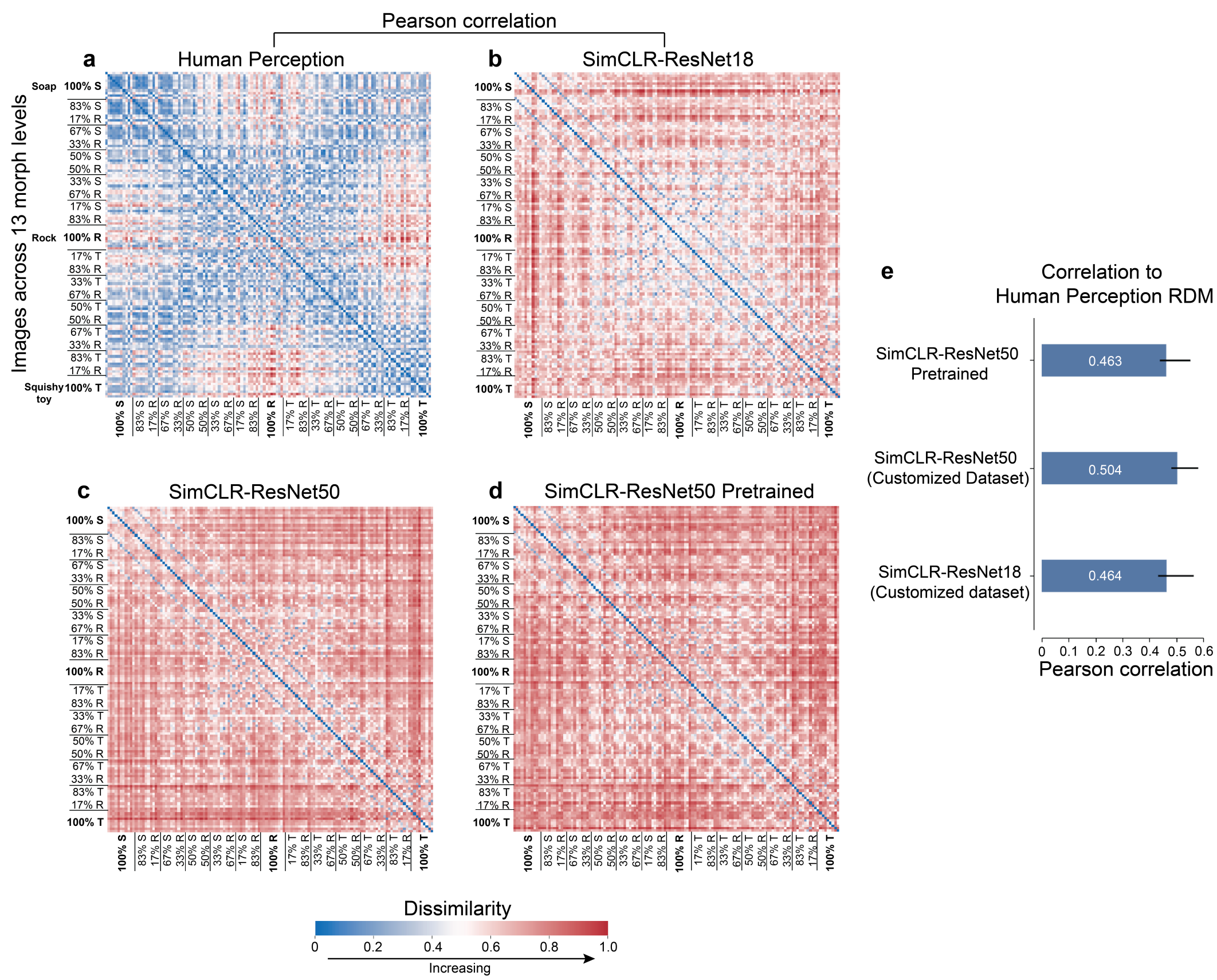

Figure S10. Comparison of model-derived representation with human perception. (a) Human perception RDM. It is constructed from pairwise Euclidean distances of five-dimensional vectors of mean property ratings. (b-d) Model-derived RDMs: Constructed from pairwise Euclidean distances between feature vectors extracted from SimCLR models (ResNet18 and ResNet50 backbones trained on our customized material image dataset; ResNet50 backbone pretrained on ImageNet-1K). The axes of all RDMs represent the 130 images, ordered by the 13 morph levels. Each RDM was normalized to 0 to 1 range. (e) Pearson correlation between the human perception RDM and model-derived RDMs. The blue bar shows the mean correlation, and the error bar indicates the 95% confidence interval via bootstrapping (1000 resampling).
